# Deep learning-based embedding of functional connectivity profiles for precision functional mapping

**DOI:** 10.1162/IMAG.a.129

**Published:** 2025-09-03

**Authors:** Jiaxin Cindy Tu, Jung-Hoon Kim, Chenyan Lu, Patrick H. Luckett, Babatunde Adeyemo, Joshua S. Shimony, Jed T. Elison, Adam T. Eggebrecht, Muriah D. Wheelock

**Affiliations:** Mallinckrodt Institute of Radiology, Washington University in St. Louis, St. Louis, MO, United States; Developing Brain Institute, Children’s National Hospital, Washington, DC, United States; Department of Neurosurgery, Washington University, St. Louis, MO, United States; Department of Psychiatry, Washington University in St. Louis, St. Louis, MO, United States; Department of Neurology, Washington University School of Medicine, St Louis, MO, United States; Institute of Child Development, University of Minnesota, Minneapolis, MN, United States; Masonic Institute for the Developing Brain, University of Minnesota, Minneapolis, MN, United States

**Keywords:** functional connectivity, resting-state fMRI, latent space, individual differences

## Abstract

Spatial similarity of functional connectivity profiles across matching anatomical locations in individuals is often calculated to delineate individual differences in functional networks. Likewise, spatial similarity is assessed across average functional connectivity profiles of groups to evaluate the maturity of functional networks during development. Despite its widespread use, spatial similarity is limited to comparing two samples at a time. In this study, we employed a variational autoencoder to embed functional connectivity profiles from various anatomical locations, individuals, and group averages for simultaneous comparison. We demonstrate that our variational autoencoder, with pre-trained weights, can project new functional connectivity profiles from the vertex space to a latent space with as few as two dimensions, yet still retain meaningful global and local structures in the data. Functional connectivity profiles from various functional networks occupy distinct compartments of the latent space. Moreover, the variability of functional connectivity profiles from the same anatomical location is readily captured in the latent space. We believe that this approach could be useful for visualization and exploratory analyses in precision functional mapping.

## 1 Introduction

Distributed large-scale networks in the human neocortex (Damoiseaux et al., 2006; M. D. Fox et al., 2005; Seitzman, Snyder, et al., 2019) with trait-like inter-individual variation in network topography studied extensively with resting-state functional MRI (fMRI) functional connectivity (FC) (Bijsterbosch et al., 2018; Braga & Buckner, 2017; Cui et al., 2020; Dworetsky et al., 2021, 2024; Glasser et al., 2016; Gordon, Laumann, Adeyemo, & Petersen, 2017; Gordon, Laumann, Adeyemo, Gilmore, et al., 2017; Gordon, Laumann, Gilmore, et al., 2017; Gratton et al., 2018; Kong et al., 2019; Kraus et al., 2021; H. Li et al., 2017; Seitzman, Gratton, et al., 2019; D. Wang et al., 2015). These functional networks have been attributed to distinct functional roles based on their strong spatial correspondence to the specialized functional systems activated during task fMRI (Cole et al., 2016; P. T. Fox & Friston, 2012; Power et al., 2011; Wig, 2017; Yeo et al., 2011). Due to the close relationship between the resting-state networks and functional significance, precision functional mapping to capture individual-specific functional networks demonstrates the potential to advance both psychiatric research and personalized therapeutic interventions (M. D. Fox et al., 2013; Gratton, Kraus, et al., 2020; Labonte et al., 2024; Lynch et al., 2022, 2024).

Many precision functional mapping methods involve the comparison of FC profiles from individual seed locations (e.g., vertices or voxels) in different brains. Reliable mapping of individual-specific functional networks requires a long fMRI data acquisition (Gordon, Laumann, Gilmore, et al., 2017). Therefore, many researchers have opted to use a group consensus network as a prior and compare the FC profiles to generate individualized functional networks. For example, one technique called “template matching” (Gordon, Laumann, Adeyemo, & Petersen, 2017; Hermosillo et al., 2024; Moore et al., 2024) assigns network identities to individual locations based on the similarity of the best-matching network average FC profiles. Another method identifies trait-like “network variants” as contiguous cortical regions with low spatial correlation between an individual FC profile and a group average FC profile from the anatomically matched seed locations (Seitzman, Gratton, et al., 2019). In a closely related school of analyses, seed-based FC profiles were compared across group-average FC data to measure FC “maturity” (i.e., similarity to adults) in pediatric cohorts (Gao et al., 2015; Sylvester et al., 2023). Despite the prevalence of using a scalar summary of the similarity between FC profiles, this does not demonstrate the specific connections that drive the similarity/dissimilarity, nor whether the number and topography of networks in the original set of network priors are appropriate. Moreover, the “network variant” and “maturity” measures only compare FC profiles with direct anatomical correspondence, yet a profound body of evidence suggests that functional correspondence across subjects may not perfectly align with anatomical correspondence.

**Fig. 1.**
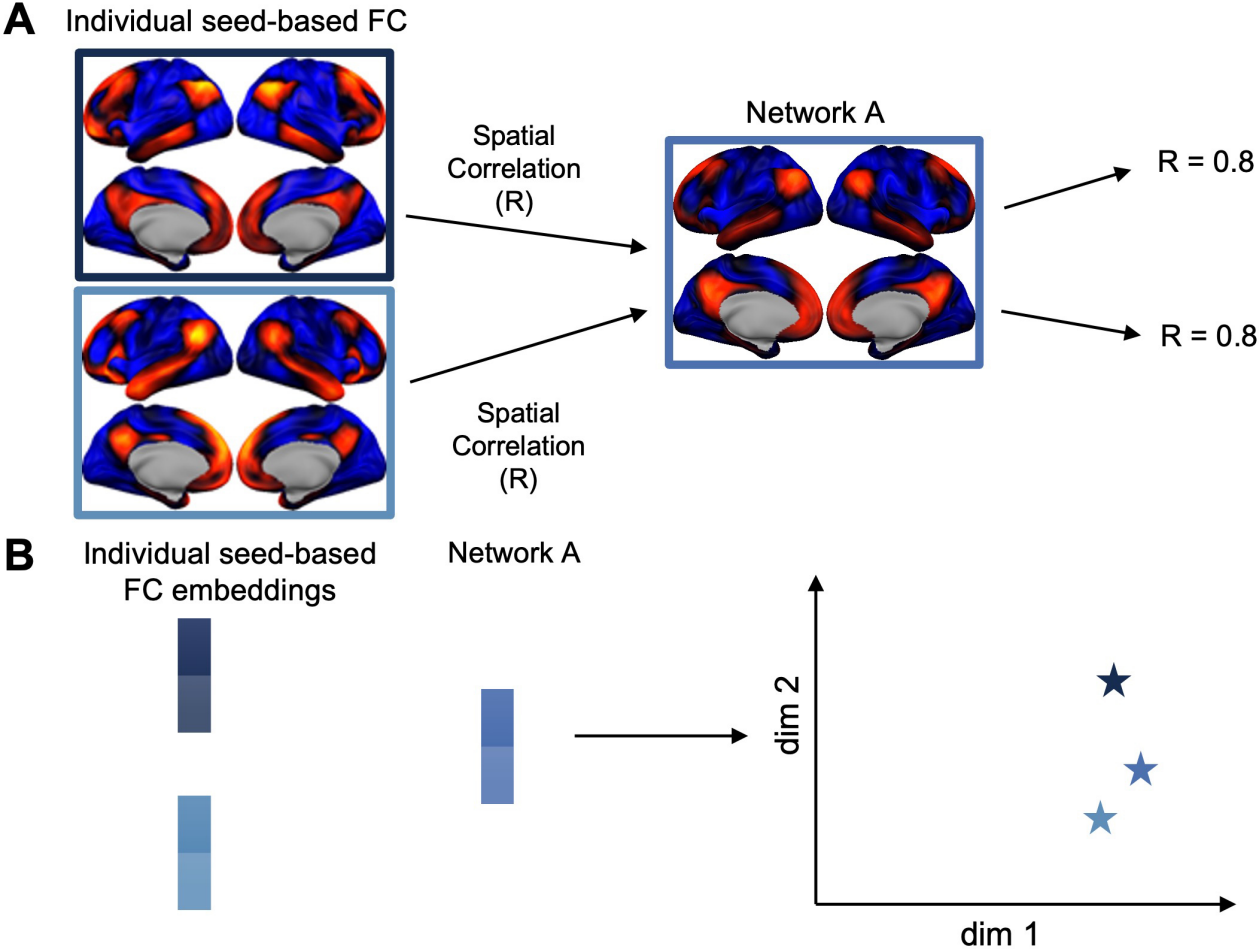
Comparing seed-based FC profiles in the vertex space versus in the latent space. (A) Vertex space: FC profiles from individual seed locations can have the spatial correlation (R) to the FC profile of a network template, despite the subtle differences between themselves. (B) Latent space: FC profiles were approximate with two-dimensional latent embeddings whose relative positions were visualized as stars.

Dimensionality reduction can be beneficial for efficiently comparing multiple samples of high-dimensional data, such as FC profiles. Various dimensionality reduction methods have been applied in neuroscience research to visualize and gain insight from high-dimensional data, including animal behavior (Stringer et al., 2019), RNA-sequencing (Zeisel et al., 2015), neural population activity (Churchland et al., 2012; Pandarinath et al., 2018), fMRI activity (Calhoun et al., 2001; Gotts et al., 2020; Kriegeskorte et al., 2008; Pospelov et al., 2021; Smith et al., 2004), and fMRI functional connectivity (Hacker et al., 2013; Margulies et al., 2016). In particular, the functional gradient calculated using diffusion map embedding on FC data has provided insights into the relative similarity of FC profiles from all seed locations in one individual or group-averaged brain in many recent studies (Dong et al., 2021; Hong et al., 2019; Langs et al., 2010, 2016; Larivière et al., 2020; Margulies et al., 2016; Nguyen et al., 2024; Tian et al., 2020; Xia et al., 2022). While most dimensionality reduction methods can effectively represent the relationship between the existing data samples in a low-dimensional latent space, few can project the data back from the low-dimensional latent space easily to the original data space, especially for new data samples outside the original data distribution. Moreover, existing methods to compare across individuals were based on first finding a low-dimensional latent space for each individual, and then applying Procrustes analysis (Bookstein, 1997) to align the spaces across subjects such that “the distance between a randomly chosen subset of vertices from the same anatomical part of the brain is minimized in functional space” (Langs et al., 2010). This kind of alignment may not produce meaningful results if the latent spaces themselves have a large disparity (Vos de Wael et al., 2020). On the other hand, generative models such as variational autoencoders (Kingma & Welling, 2013) can generate new data points and provide a bidirectional mapping between the data space and the latent space. Recently, a beta-variational autoencoder has been introduced for the automated discovery of interpretable factorized latent representations in images (Higgins et al., 2017), and has been used for disentangling resting-state fMRI activity in both adults (Kim et al., 2021) and fetuses/neonates (Kim et al., 2023). Here, we introduce the use of a variational autoencoder to disentangle interpretable factors driving the variation in FC profile across both locations and subjects in a low-dimensional latent space. One toy example contrasting the comparison of FC profiles in vertex space and a low-dimensional latent space is provided in Figure 1A.

## 2 Methods

### 2.1 Neuroimaging datasets

We used resting-state fMRI data from the Washington University 120 (WU120) (Power et al., 2014) to train the model weights to project the FC profiles from the original vertex space to a low-dimensional latent space. The WU120 dataset contains one resting-state session per subject for 120 subjects. Out of the 120 subjects from WU120, 100 subjects were selected as the training data, 10 as the validation data, and 10 as the test data. For additional analyses based on the pre-trained weights, we utilized the resting-state fMRI data from the Human Connectome Project (HCP, Rest1, and Rest2 scan sessions in 94 unrelated individuals) (Van Essen, Ugurbil, et al., 2012), the Midnight Scan Club (MSC, 10 scan sessions in 10 individuals) (Gordon, Laumann, Gilmore, et al., 2017), and Baby Connectome Project (BCP, 301 scan sessions in 178 individuals) (Howell et al., 2019) datasets. All datasets used are publicly available, and the paths to the data are provided in the “Data and Code Availability” section.

The adult datasets (all except BCP) were collected from young adult subjects (19–35 years) in a 3-Tesla MRI scanner while they were asked to fixate on a center cross on the screen. The baby dataset (BCP) was collected from infants, toddlers and preschool children (8–60 months) during natural sleep. Procedures were then applied to normalize intensity, correct for motion in the scanner, and transform the data onto a standard 32k-fsLR surface (Van Essen, Glasser, et al., 2012). Furthermore, motion and other non-neuronal sources of artifact were mitigated with nuisance regression (including global signal regression) and bandpass filtering (Power et al., 2014) in the volume data before transforming to the surface space (adult datasets), or after transforming to the surface space (baby dataset). Further details on the acquisition and processing of the neuroimaging datasets and subject demographics are available in the Supplementary Materials.

### 2.2 Functional connectivity (FC) profiles

The FC profile from each seed vertex was calculated as the Pearson’s correlation between the BOLD time series from that vertex to all cortical vertices in the left and right hemispheres (N = 59412 in the standard 32k-fsLR surface, after masking out medial wall). FC profiles from a randomly sampled 10% of the vertices in each of the 100 subjects were used as the samples (N = 5942 x 100 = 594200) for training the model weights to map data from the vertex space (59412 dimensions) to the latent space to balance between the variability in the training samples and computational demand.

In addition, we also calculated the FC profiles from individual area parcels (N = 333 for the group-average adult parcellation (Gordon et al., 2016), N = 567–710 for Midnight Scan Club individual-specific parcellations (Gordon, Laumann, Gilmore, et al., 2017), and N = 326 for the group-average toddler parcellation (Tu et al., 2025)), where each area consists of tens to hundreds of vertices. The FC profile from each area was calculated with Pearson’s correlation between the average BOLD time series from each area and the BOLD time series from each of the 59412 vertices. FC profiles from functional networks (each with thousands of vertices) and the functional network prior (average FC profiles for functional networks across subjects) can be calculated with the same logic.

Unless stated otherwise, the area parcellations and the FC profiles were visualized on a group-average brain surface in the standard 32k-fsLR space based on the MNI or Conte69 templates (Brett et al., 2002; Glasser & Van Essen, 2011).

### 2.3 The variational autoencoder model

Autoencoders (AE) are neural networks designed to encode the input into a compressed representation, and then decode it back to a reconstructed input similar to the original one. The variational autoencoders (VAE) (Kingma & Welling, 2013) learn a distribution in the compressed representation. They are especially useful in obtaining a smooth, continuous latent space for generating new data, with the power to disentangle latent generative factors from images further enhanced with a higher weight on the Kullback-Leibler (KL) divergence in the cost function with a hyperparameter β (Higgins et al., 2017). Unlike diffusion map embedding, AEs feature an encoder and decoder design for straightforward application to embed new data and reconstruct latent embeddings in the original vertex space. We adopted the same model architecture as described in prior research (Kim et al., 2021, 2023, 2024) with five convolutional layers and one fully-connected layer in the encoder, and one fully-connected layer and five convolutional layers in the decoder.

**Fig. 2.**
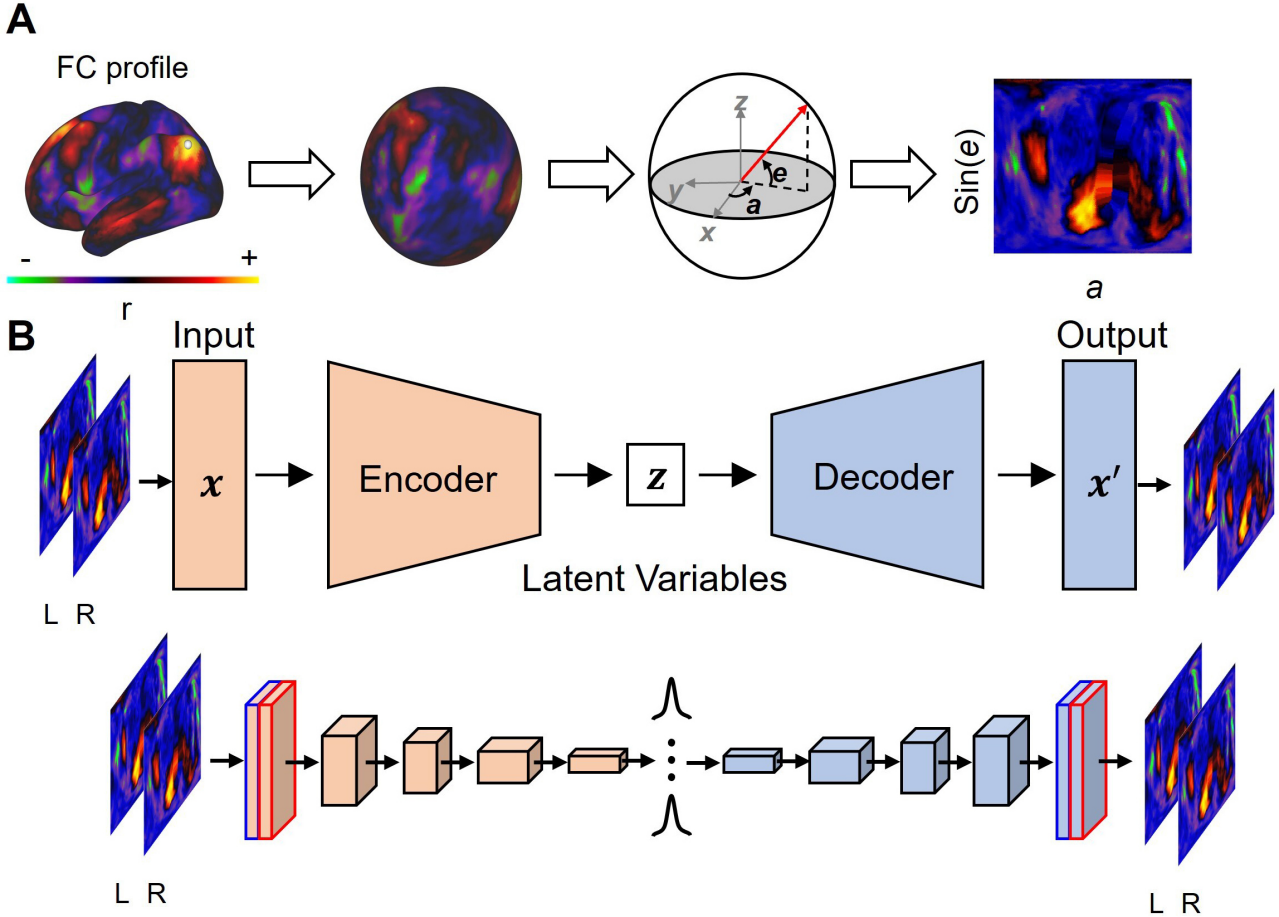
Geometric reformatting and the autoencoder model architecture. (A) Geometric reformatting. The cortical distribution of fMRI activity is converted into a spherical surface and then to an image by evenly resampling the spherical surface with respect to sin(e) and a, where e and a indicate elevation and azimuth, respectively. (B) Architecture of an autoencoder. An encoder network samples latent variables given an input image under the inference model, while a decoder network generates a genuine input image under the generative model. Both the encoder and decoder networks contain 5 convolutional layers. Adapted from Kim et al. (2021). Copyright 2021 by Elsevier Inc.

To take advantage of the convolutional layers in efficiently representing local patterns in images with few weights, we formatted the FC profiles into surfaces to 2D images. The geometric reformatting procedure was done in four steps. First, the FC profiles were mapped to the cortical surface using their coordinates in the 32k-fsLR mesh of the left and right hemispheres (32492 vertices per hemisphere, with some of them empty due to the presence of the medial wall). Then, the surfaces in each hemisphere were inflated to a sphere using FreeSurfer (Fischl, 2012). After that, we used cart2sph.m in MATLAB to convert its Cartesian coordinates (x,y,z) to spherical coordinates (a,e), which reported the azimuth and elevation angles in a range from −π  to +π and from −π​/​2  to +π​/​2 , respectively. Lastly, we defined a 192 × 192 grid to resample the spherical surface with respect to azimuth and sin(elevation) such that the resampled locations were uniformly distributed at approximation (Fig. 2A).

The encoder transformed an FC profile (a pair of left and right hemisphere images formatted to two 192 x 192 grids) into a probabilistic distribution of N latent variables, where N is the number of latent dimensions. For visualization purposes, we use N = 2, although we have conducted additional experiments using different numbers of dimensions in Supplementary Materials (Section C). Each convolutional layer conducted linear convolutions followed by rectifying the outputs as described by Nair and Hinton (2010). The first layer utilized 8 × 8 convolutions on inputs from each hemisphere and combined the results. Subsequent layers, from the second to the fifth, applied 4 × 4 convolutions to this combined output. Circular padding was employed at the azimuth boundaries, while zero padding was used at the elevation boundaries. A fully connected layer applied linear weighting to generate the mean and standard deviation for the distribution of each latent variable. The decoder replicated this structure in reverse, reconnecting the layers to recreate the FC profile from a sample latent variable. The VAE model was optimized to reconstruct the input while constraining the distribution of every latent variable to be close to an independent and standard normal distribution. This is achieved through optimizing the encoding parameters, ϕ, and the decoding parameters, θ, to minimize the loss function below:

$$L(\phi ,\theta |x)\;=∥x-x^{\prime }{∥}_{2}\;+\;\beta \cdot D_{KL}[N(\mu_{z},\sigma_{z})||N\left(0,I\right)]$$ (1)

where x is the input data from both hemispheres, $x^{\prime }$ is the reconstructed data, and $N(\mu_{z},\sigma_{z})$ is the posterior distribution, N(0,I) is the prior distribution. $D_{KL}$ measures the KL divergence between the posterior and prior distributions, and β is a hyperparameter balancing the two terms in the loss function. A β < 1 places less emphasis on the KL divergence and focuses more on reconstruction, while a β > 1 places a higher emphasis on KL divergence, enforcing stricter regularization of the latent space.

Models were trained with stochastic gradient descent with a batch size of 128, an initial learning rate of 1E-4, and 50 epochs with random data selection in each batch. An Adam optimizer (Kingma & Ba, 2014) was implemented, and the learning rate decayed by a factor of 10 every 20 epochs. Final hyperparameters, including the number of latent dimensions and the beta value, were determined by the trade-off between KL divergence and reconstruction loss on the validation data. The model was trained in Python 3.8 using PyTorch (v2.1.2 + cu118) using a server with an NVIDIA A100 GPU (40GB memory).

Additional details on the model design and hyperparameter tuning are provided in the Supplementary Materials (Section B). We also briefly explored alternative dimensionality reduction methods in the Supplementary Materials (Section D).

### 2.4 Quality of clustering by functional networks

Areas within the same functional network tend to possess similar FC profiles (Yeo et al., 2011). The 286 out of 333 area parcels in the Gordon parcellation (Gordon et al., 2016) were grouped into 12 functional networks. The additional 47 parcels that fell in the low-SNR regions and cannot be confidently grouped into any of the functional networks (with “None” network assignment in the original paper) were excluded from further analyses. We evaluated the segregation of FC profiles from different functional networks with the silhouette index (SI) (Rousseeuw, 1987; Yeo et al., 2011) on the group-average FC profiles or group-average latent embeddings. The SI is calculated as:

$$SI\left(i\right)\;=\;\frac{b_{i}-a_{i}}{max\left(a_{i},b_{i}\right)}$$ (2)

where $a_{i}$ is the mean within-network distance, and $b_{i}$ is the smallest mean between-network distance to alternative networks. A correlational distance measure was used for the FC profiles in the vertex space, whereas a Euclidean distance measure was used for the FC profiles in the latent space. A 95% confidence interval (95% CI) was calculated by bootstrapping the subject samples 1000 times.

### 2.5 Between-subject and within-subject variability

The between-subject variability in the latent embeddings for a given parcel can be quantified with an inter-subject “dispersion” measure (Alberti et al., 2025), where dispersion was calculated as the mean of squared Euclidean distances from parcel embeddings across different subjects to the across-subject centroid. Similarly, the within-subject variability can be calculated as an intra-subject dispersion with the two repeated sessions Rest1 and Rest2 in the HCP data (N.B. we used mean instead of sum of squared Euclidean distances, unlike Alberti and colleagues, so that the inter-subject and intra-subject dispersion values are comparable despite the differences in number of samples). A dispersion signal-to-noise (SNR) was calculated to quantify the relative magnitude of inter-subject and intra-subject dispersion of the embeddings.

### 2.6 Test for individual specificity

One way of measuring individual specificity in functional connectome is the “fingerprinting” analysis (Finn et al., 2015). Since the parcel-wise connectome matrix (333 x 333) is symmetric, we concatenated the lower triangle into a vector as a “barcode” for the first and second sessions, respectively. The similarity across sessions for the same and different subjects was calculated using Pearson’s correlation between those “barcodes”. For the latent representations with dimensions 2, 4, and 32, we concatenated the values from all dimensions for each session as the “barcode” for the session and repeated the fingerprinting analysis. The average accuracy of identification was calculated as the average of two numbers: the proportion of correct identification of the subject from the session 1 data, and the proportion of correct identification from the session 2 data.

Another way to examine individual specificity is to test whether individuals’ demographic features could be predicted by the FC. Previous studies have found great age-related variance in FC in the BCP data (Kardan et al., 2022), so we used the BCP data for this analysis. The low-dimensional latent embeddings from VAE (326 × zdim features, where zdim = 2, 4, 32) or 52975 unique features (upper triangle from 326 × 326 parcels) were used as features. We used a support vector machine regression for age, with 80% of the data used for training and the remaining 20% used for testing. We applied a ridge regularization where the hyperparameter lambda was optimized with a 5-fold cross-validation approach from a range of 15 lambda values from $10^{-8}$ to $10^{3}$ evenly distributed on a log scale. This process was repeated 1000 times by randomly splitting training and test data, and the mean and standard deviation of the age prediction performance, measured with Pearson’s correlation (r), were calculated for each sample. We also calculated the same metric from the other dimensionality reduction methods, reported in Supplementary Materials (Section D).

### 2.7 Relationship between functional profiles and intelligence

To examine how variance in each dimension of the latent space is related to variance in individual cognitive traits, we correlate the fluid intelligence and crystallized intelligence scores with the two VAE latent embedding values of each parcel. To obtain enough power for this analysis, we use the 965 HCP individuals with at least 10 minutes of low-motion data in both Rest1 and Rest2, concatenate both Rest1 and Rest2 sessions to calculate the FC, and obtain the latent embeddings of FC in each parcel. Pearson’s correlation between the FC latent embedding of each dimension and 1) fluid intelligence composite score (mean = 115.6, SD = 11.5, missing 12 values) and 2) the crystallized intelligence composite score (mean = 118.0, SD = 9.8, missing 5 values) were calculated for each parcel. For each dimension-intelligence score pair, the p-values were adjusted by the linear-step up procedure originally introduced for false discovery rate (FDR) correction by Benjamini and Hochberg (1995).

## 3 Results

### 3.1 A traversal through the latent space gives rise to systematic variations in reconstructed functional connectivity profiles

**Fig. 3.**
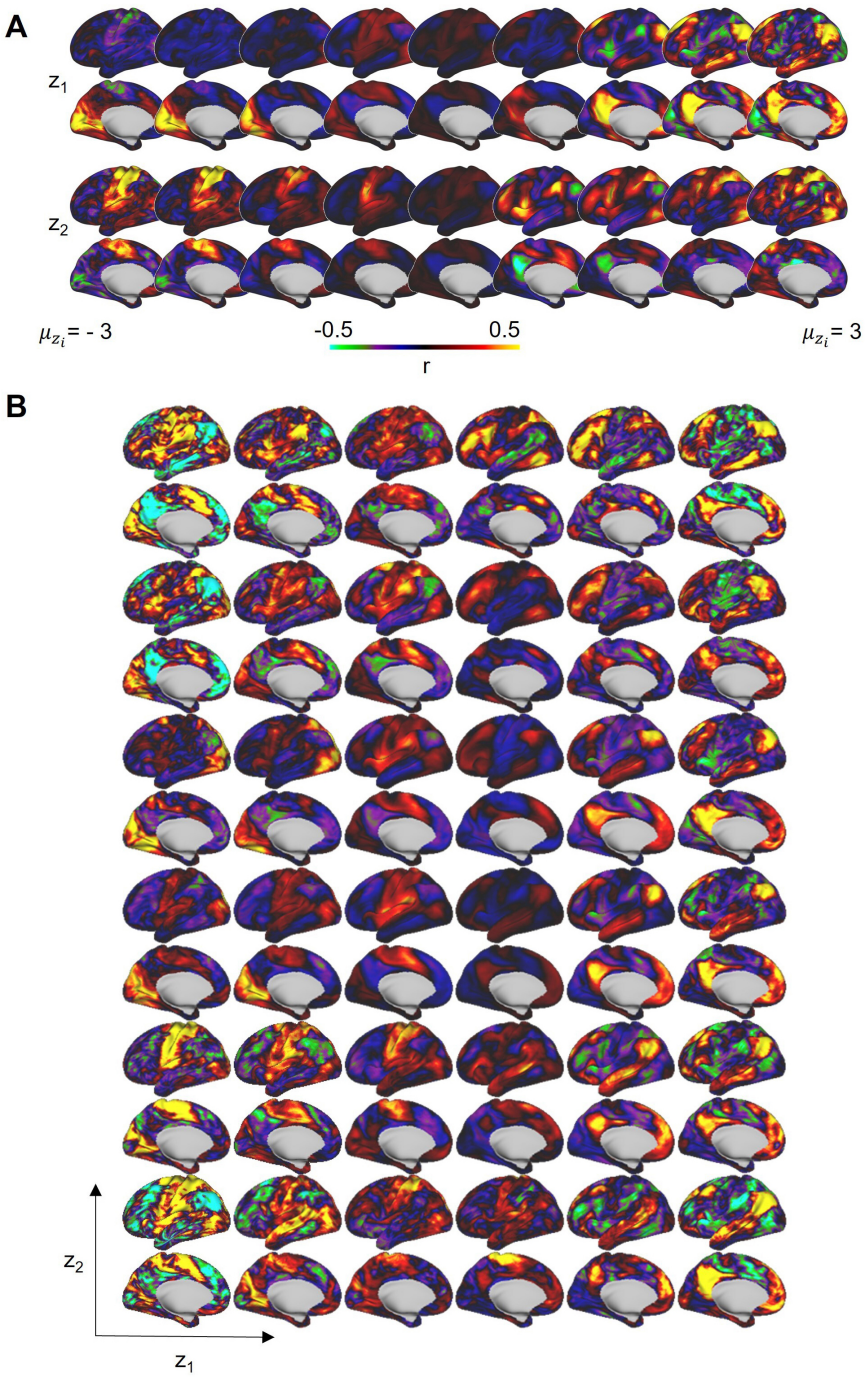
Reconstructed FC profiles obtained by traversing the latent space of the beta-VAE model. (A) One latent dimension varies in equal steps from one end to the other, while the other dimension is fixed at zero. (B) Grids representing different combinations of z_1_ and z_2_.

To understand how the changes in the magnitude of each latent dimension affect the reconstructed FC profile appearance, we evenly divided one of the two latent dimensions (z_1_ and z_2_) while keeping the other latent dimension fixed at zero, and then back-projected those latent embeddings to the vertex space using the VAE decoder. We observed patterns reminiscent of sensorimotor networks and association networks (Margulies et al., 2016; Sydnor et al., 2021), as well as task-positive to task-negative networks (Buckner et al., 2008; M. D. Fox et al., 2005; Raichle, 2015) (Fig. 3A). Furthermore, somatomotor hand versus mouth and visual versus somatomotor network separations were also observable, suggesting the disentanglement of not only the coarse separation mentioned above but also fine details within the sensorimotor networks. Additionally, we obtained realistic FC profiles commonly observed across different functional networks for combinations of z_1_ and z_2_ (Figure 3B).

### 3.2 Functional connectivity profiles are internally coherent within functional networks in both the vertex space and the latent space

**Fig. 4.**
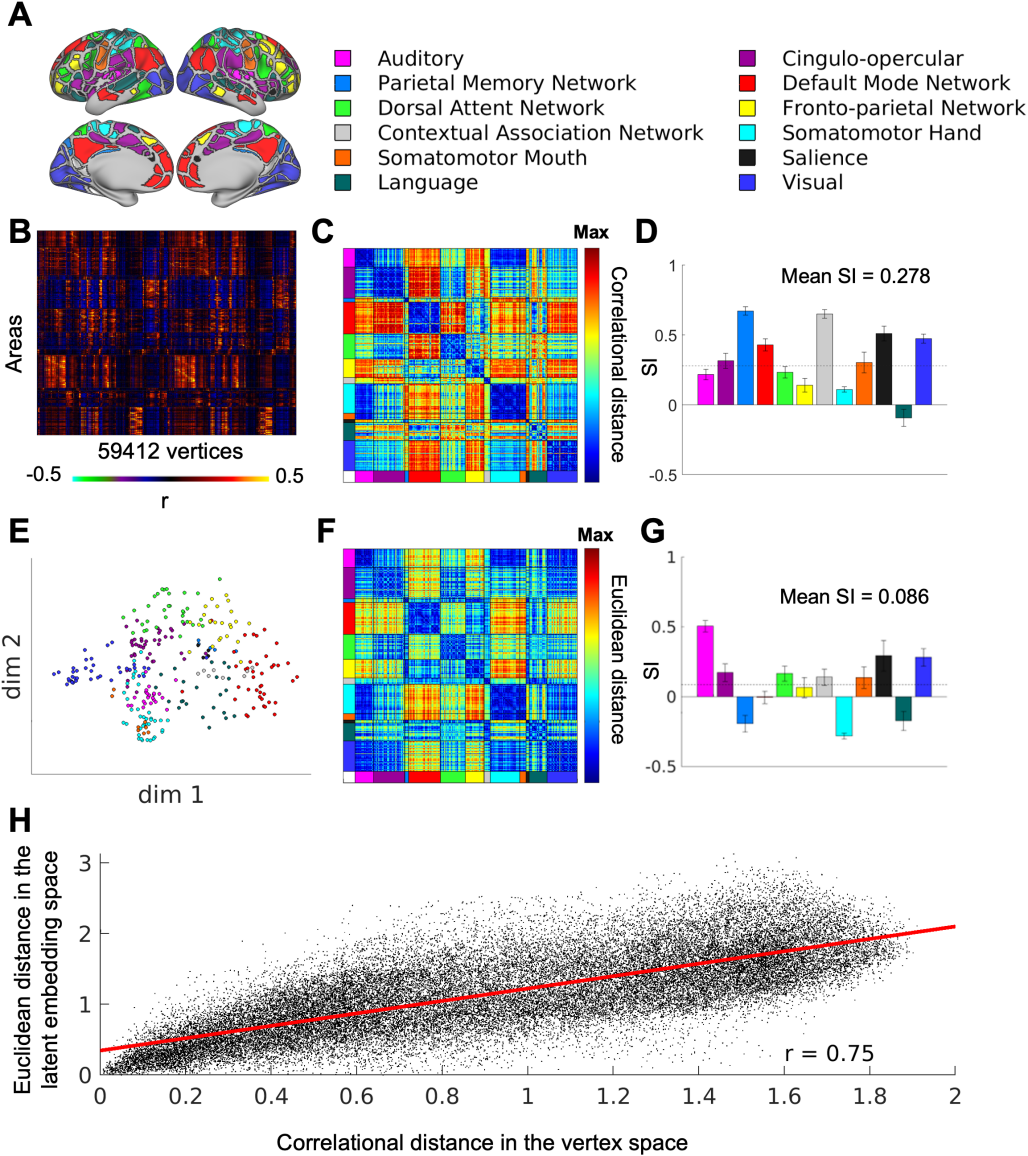
Separation of functional connectivity (FC) profiles by functional networks in the average of 94 HCP subjects. (A) Gordon network assignments for 286 area parcels (some networks were renamed from the original publication based on later discovery of domain functions). (B) The flattened FC profiles from each of the 286 area parcels. (C) The mean correlational distance between each pair of the FC profiles in B. (D) The mean silhouette index for each functional network based on the correlation distance in C. The dashed line shows the mean across all areas. (E) The FC profile latent embeddings with two dimensions in VAE. Each circle represents each area parcel’s mean functional connectivity profile across Rest1 sessions of 94 subjects. (F) The mean Euclidean distance between the latent embeddings of the average across 94 subjects. (G) The mean silhouette index for each functional network based on the Euclidean distance in F. The dashed line shows the mean across all areas. (H) A scatter plot of the Euclidean distance in the latent space (F) and correlational distance in the vertex space (C).

It has been well established that FC profiles from the same functional networks tend to be similar (Buckner et al., 2013; Yeo et al., 2011). In this study, we validated this observation in the 94 unrelated subjects from the HCP dataset and evaluated whether similar patterns could be detected in the latent embeddings. When sorting the group-average FC profile from each of the 286 areas within the 12 networks defined by the Gordon parcellation (Gordon et al., 2016) according to the network order (Fig. 4A), we observed that FC profiles within the same network appeared qualitatively similar (each row in Figure 4B is a flattened FC map from the 59412 cortical vertices). The relative similarity between the FC profiles (i.e., rows in Fig. 4B) can be quantified using correlation distance (1-Pearson’s correlation). We found that within-network distances tend to be smaller than between-network distances (Fig. 4C). On average, the FC profile of each area was more similar to those within the same network than to those in the closest alternative network (mean Silhouette Index (SI) > 0) (Fig. 4D). When projected onto a two-dimensional latent space, the FC profiles form clusters that are closer together within the same functional networks, based on Euclidean distances (Fig. 4E, F). On average, the FC embeddings from each area were closer to those within the same network than to those in the nearest alternative network (mean SI > 0). However, the mean SI was lower than previously observed in the vertex space, and some networks, such as the default mode network (red), were not distinctly separated from their closest alternative network. We will explore this observation further in the next section. Additionally, we found that the mean SI first increased and then decreased with the number of latent dimensions, potentially due to the “curse of dimensionality,” where all points are far apart at higher dimensions (Supplementary Materials—Section C). For completeness, we compare our results to other linear and non-linear dimensionality methods: at dimensionality = 2, the reconstruction performance was higher for the nonlinear methods, including variants of autoencoders, than for linear methods, including principal component analysis (PCA) and independent component analysis (ICA). The different sensorimotor networks were also better separated in our VAE model than the PCA and ICA models (Supplementary Materials—Section D). We also repeated the same analysis on the fMRI data collected during the Working Memory task in the same subjects and observed task-rest differences in the distribution of FC embeddings from the whole-brain (Supplementary Materials—Section E).

### 3.3 Functional connectivity profiles in the default mode network were separated into sub-networks in the low-dimensional latent space

**Fig. 5.**
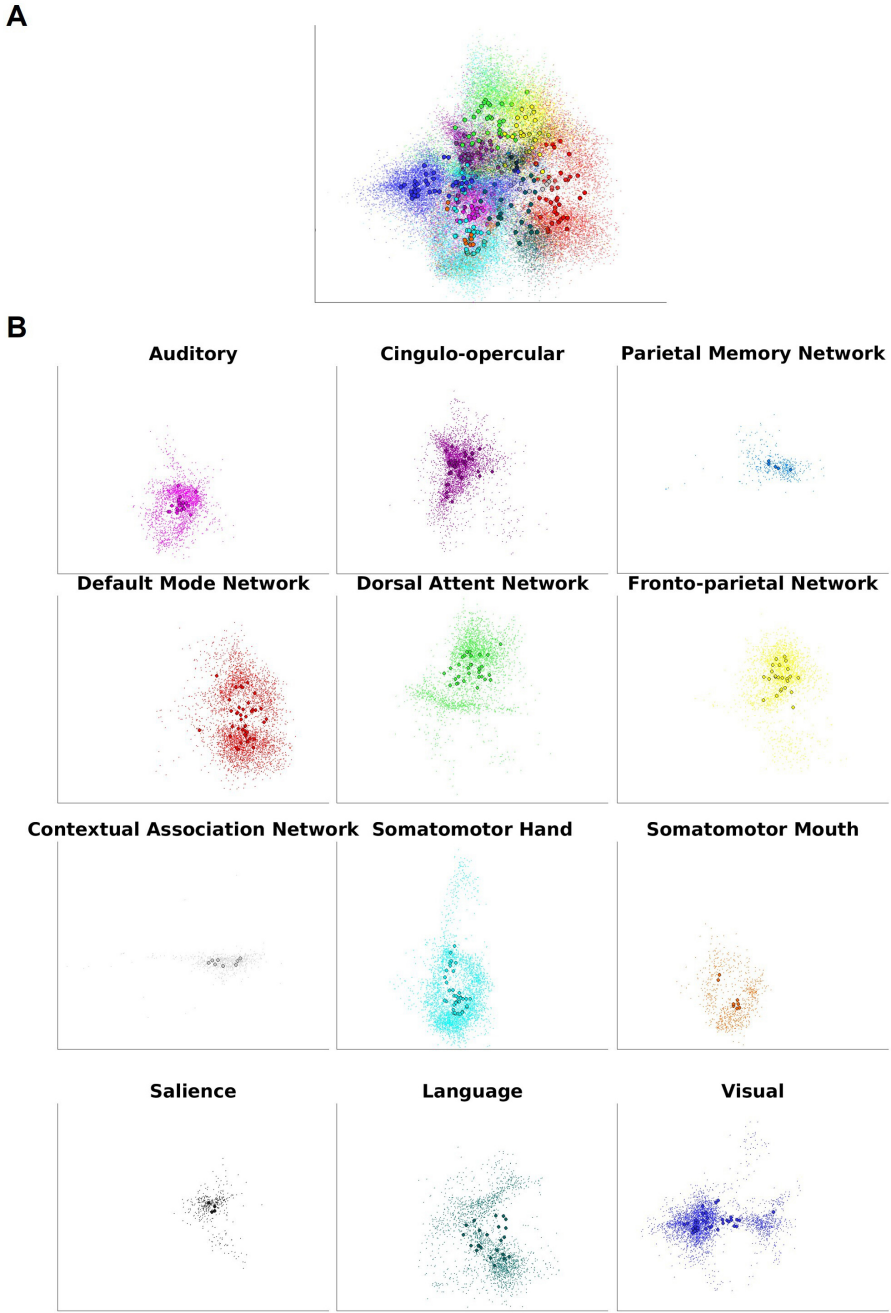
FC profile embeddings from the Rest1 session for areas within the 12 Gordon networks across all 94 HCP subjects. (A) All 12 networks. (B) Each individual network. The large circles demonstrate the average of each parcel across subjects, and the smaller dots represent individual parcels from individual subjects.

**Fig. 6.**
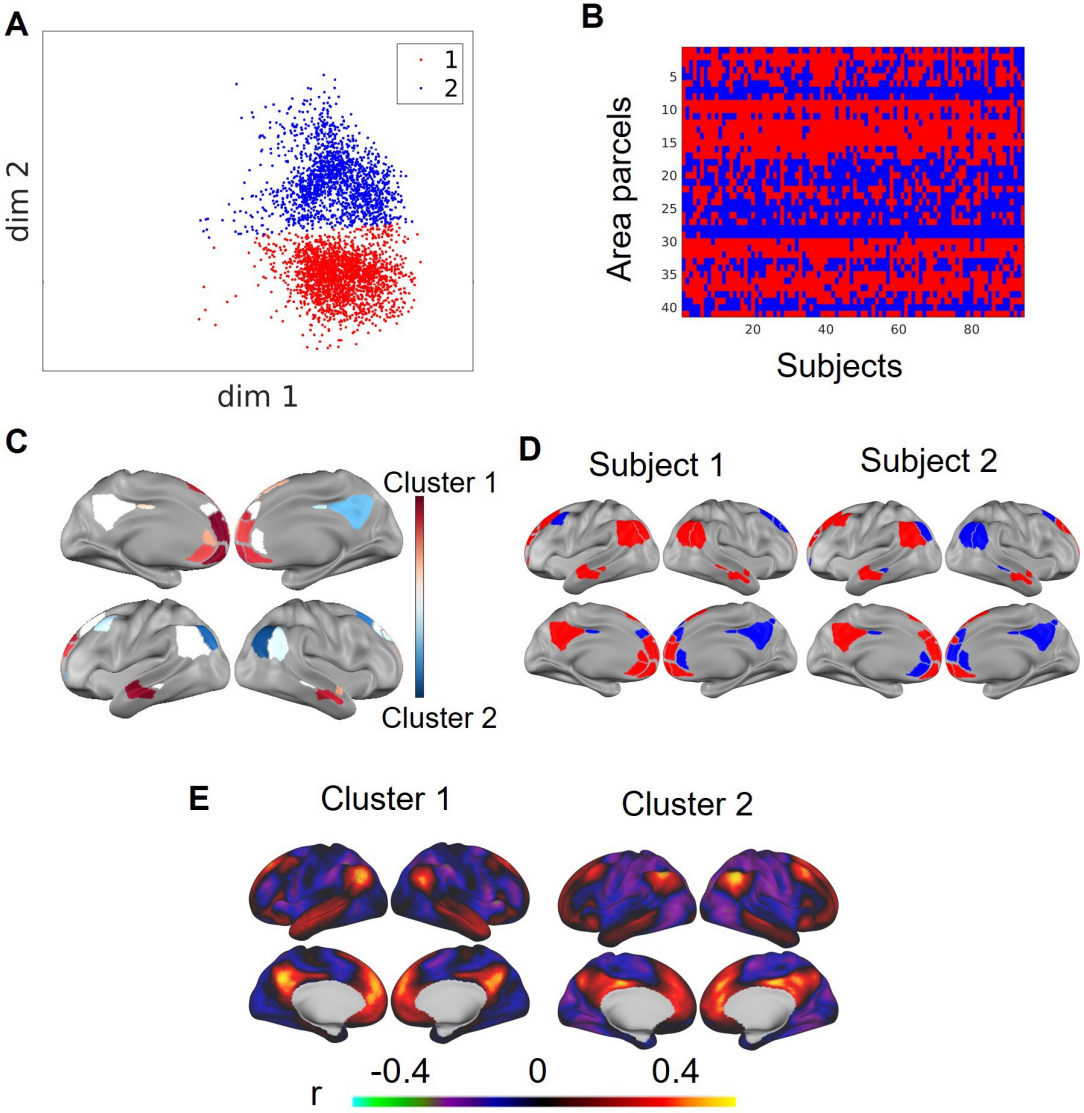
Sub-clusters within the DMN network in the latent space. (A) Clustering the DMN latent embeddings into two sub-clusters with the k-means algorithm. (B) The sub-cluster membership across parcels and subjects. (C) The probability of each parcel belonging to each cluster is calculated as a percentage of the population. (D) The sub-cluster membership in two example subjects. (E) The reconstructed FC profiles from the centroids of the two sub-clusters. The axes scales were the same as Figure 5.

In addition to the group average, we can also examine the variability across the functional networks across all area parcels from the 94 subjects in the HCP dataset (Fig. 5). It appears that while FC profiles from some networks form relatively local and spherical clusters in the latent space (e.g., the contextual association, salience and parietal memory networks), other networks exhibit more complex shapes and are sometimes divided into multiple spherical clusters across all subjects (e.g., default mode network (DMN)). To further investigate whether these clusters correspond to biologically meaningful divisions, and whether the sub-clusters correspond to the clustering within subjects or sub-networks, we applied k-means clustering to the FC profiles from areas belonging to the default mode network from all subjects, choosing k = 2 based on visual inspection (Fig. 6A, B). The resultant cluster consists of data from similar area parcels across the subjects (Fig. 6C), albeit with some variability (Fig. 6D). Cluster 1 primarily includes parcels in the medial prefrontal cortex, the temporal cortex and the dorsolateral prefrontal cortex, while Cluster 2 is mainly composed of parcels in the inferior parietal cortex and the posterior cingulate cortex (Fig. 6E). Subtle differences can be observed in the reconstructed FC profiles from the centroids of the two clusters (Fig. 6F). The reconstructed FC profile from the centroid of Cluster 1 resembles the ventromedial, pregenual and parietal components of the default network, while the distribution of Cluster 2 mirrors the dorsolateral and retrosplenial components of the default network. Similar division of the default mode subnetworks has been identified in other studies (Akiki & Abdallah, 2019; Andrews-Hanna et al., 2010; Gordon et al., 2020; Lynch et al., 2024; Uddin et al., 2009).

### 3.4 Interindividual variability in functional connectivity profiles from the same anatomically-matched location was evident in the low-dimensional latent space

**Fig. 7.**
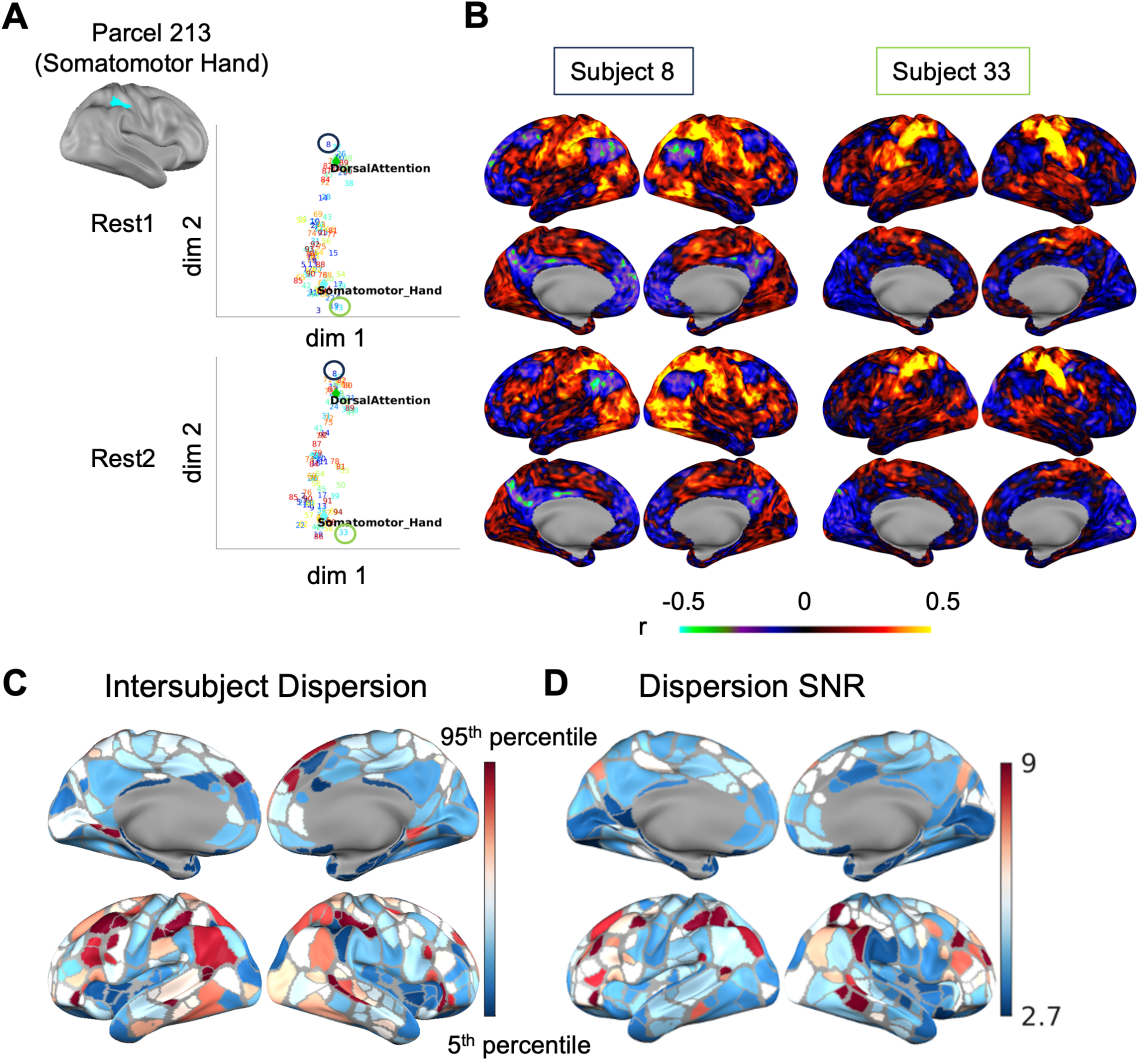
Interindividual variability in each parcel’s FC embedding across 94 HCP subjects in the VAE latent representation in Rest1 and Rest2 sessions. (A) The FC embedding for example parcel 213. Each data point is a subject indicated by the numbers 1 to 94. The “Somatomotor_Hand” and “DorsalAttention” markers were from Lynch et al. 2024. The axes scales were the same as Figure 5. (B) The FC profiles from parcel 213 for example subjects 8 and 33 in Rest1 and Rest2 were visualized on standard brain surfaces. (C) Intersubject dispersion in the Rest1 sessions. (D) Dispersion signal-to-noise ratio (SNR) of each parcel’s FC calculated as the ratio of intersubject dispersion and intrasubject dispersion.

Despite the largely consistent topography of functional networks across adult individuals (Damoiseaux et al., 2006; Gratton et al., 2018), interindividual differences in functional network assignment have been well documented in prior studies (Bijsterbosch et al., 2018; Braga & Buckner, 2017; Cui et al., 2020; Dworetsky et al., 2021, 2024; Glasser et al., 2016; Gordon, Laumann, Adeyemo, & Petersen, 2017; Gordon, Laumann, Adeyemo, Gilmore, et al., 2017; Gordon, Laumann, Gilmore, et al., 2017; Gratton et al., 2018; Kong et al., 2019; Kraus et al., 2021; Langs et al., 2016; H. Li et al., 2017; Seitzman, Gratton, et al., 2019; D. Wang et al., 2015). Here, we examine the position of the FC profile embedding from one example area parcel assigned to the “Somatomotor Hand” network based on the Gordon parcellation using group-average adult data (Gordon et al., 2016). The variability of FC profiles from this example parcel was substantial (Fig. 7A, the scales of the axes were the same as Figs. 5 and 6). Additionally, the relative positions of the embeddings for different subjects were qualitatively consistent across two resting scan sessions (Rest1 and Rest2) in the VAE latent space (Fig. 7A). The original FC profiles on the brain revealed that the two subjects at the extremes of the distribution demonstrated very distinct FC profiles: one resembling the “Dorsal Attention” network and the other “Somatomotor Hand” network (Fig. 7B). Intersubject dispersion tends to be higher on the temporal, parietal, and frontal cortices (Fig. 7C). Similarly, we found that the association cortices (frontal and parietal cortices) tend to have a higher ratio of intersubject dispersion to intra-subject dispersion than sensorimotor cortices (Fig. 7D).

**Fig. 8.**
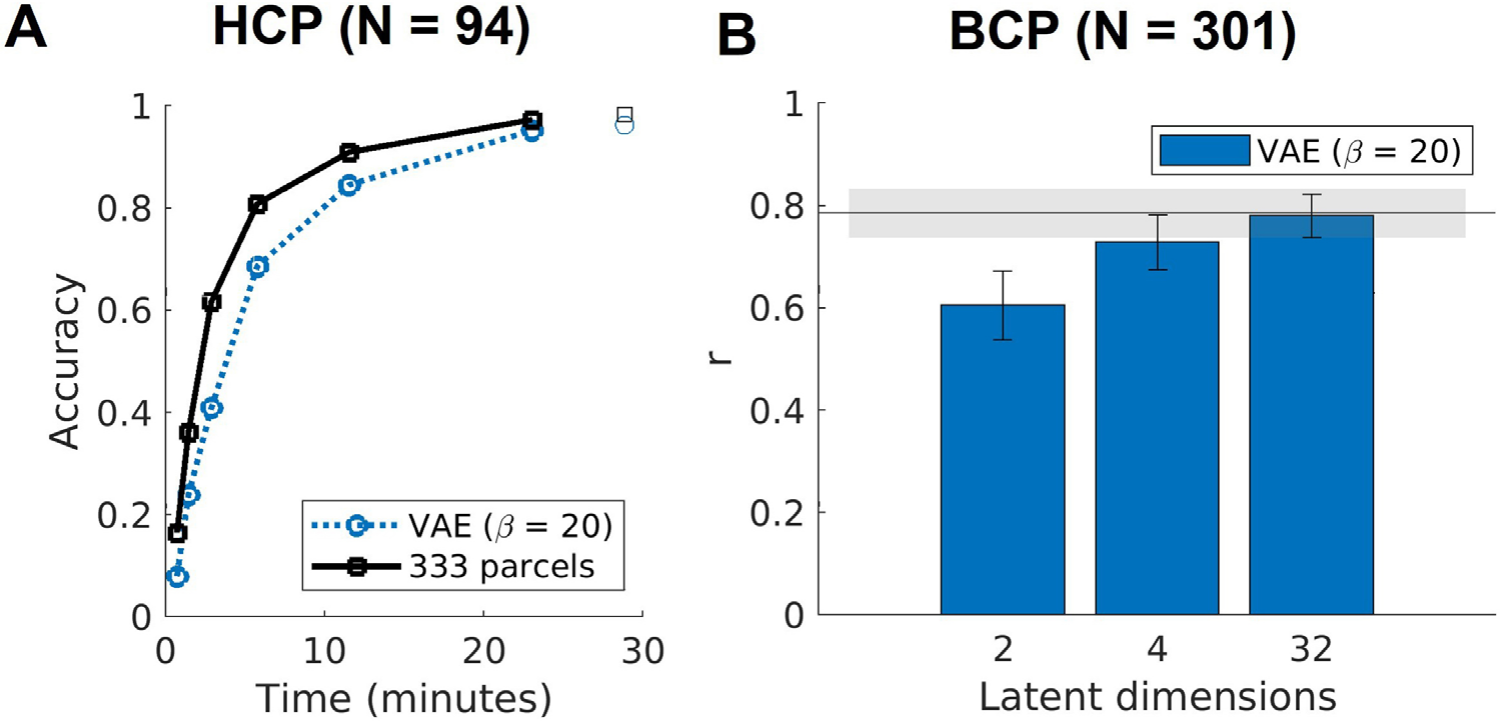
Fingerprinting accuracy and age prediction. (A) The lines show fingerprinting performances from increasing amounts of data in each session, and the isolated markers show the fingerprinting performance from the whole session in 94 HCP individuals. (B) Age prediction accuracy is measured as the Pearson’s correlation (r) between the actual and predicted age in years for 301 BCP sessions. Error bars show the mean and standard deviation across the 1000 samples. The horizontal line and shaded area show the performance (mean and standard deviation) using features in the parcel space.

In addition to examining the interindividual variability in FC from a single parcel, we also quantified the interindividual variability in FC from all parcels. We used connectome fingerprinting to test whether different sessions of the same individual can be successfully identified from a list of subjects (Finn et al., 2015) using the 94 unrelated HCP subjects. The accuracy of fingerprinting increased with the amount of data (no motion frame censoring was applied for this analysis). While the fingerprinting accuracy from the latent embeddings was marginally lower than the parcel connectome with fewer data, the performance was similar with a relatively long scan (>20 minutes) (Fig. 8A). This further supported the idea that interindividual variability is preserved by the low-dimensional embeddings. Results from other dimensionality reduction methods and latent dimensionality are similar, with VAE (β =20)  with the top performance and fingerprinting accuracy increasing with the number of dimensions—Supplementary Materials (Section D.5). It is worth noting, though, that our goal was not to maximize the identifiability of the whole connectome as outlined in previous studies (Cai et al., 2021), but rather to provide intuition of the variability of seed-based FC from single seeds across both seed locations and individuals.

Another way to demonstrate the interindividual variability would be to predict individual demographic characteristics, such as chronological age. Since the age-related variance is largest in younger subjects, we used the 301 sessions from the BCP data for this analysis. The mean Pearson correlation between predicted and true age across 1000 train-test splits was 0.6061 (SD = 0.0668, 95% CI [0.4585, 0.7256]) for dimension = 2, 0.7291 (SD = 0.0537, 95% CI [0.6196, 0.8270]) for dimension = 4, 0.7806 (SD = 0.0425, 95% CI [0.6964, 0.8574]) for dimension = 32, and 0.7858 (SD = 0.0480, 95% CI [0.6744, 0.8630]) for dimension = 326 parcels (Fig. 8B). We found qualitatively similar results across alternative dimensionality reduction methods, where age-prediction performance was higher for higher latent dimensions (Supplementary Fig. D.6).

**Fig. 9.**
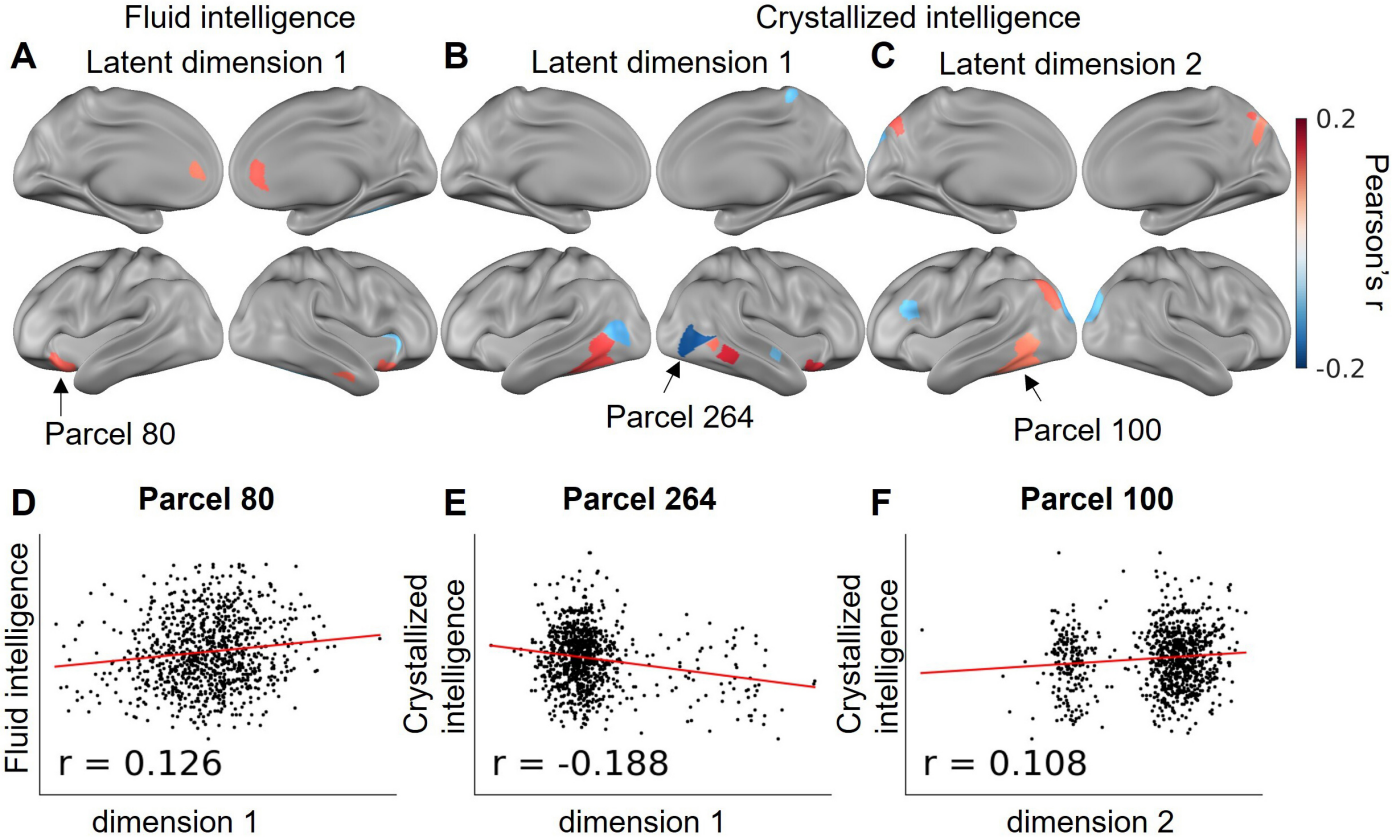
Relating cognitive traits to FC latent embeddings. (A–C) Parcels with significant correlation with intelligence composite scores. (D–F) Scatter plot of the intelligence composite scores and the latent embedding values of dimension 1 or dimension 2 of an example parcel.

One hypothesis is that the interindividual variability in FC latent embedding position captures the subjects’ differences in cognitive traits (Alberti et al., 2025). We correlated the latent embedding values for each dimension of the whole set of HCP subjects with more than 10 minutes of low-motion data in both Rest1 and Rest2 sessions (N = 965, details in Section 2.7). After FDR-correction, the first latent dimension from 8 parcels had a significant correlation (4 positive and 4 negative) with the fluid intelligence composite score (Fig. 9A), the first latent dimension from 8 parcels had a significant correlation (5 positive and 6 negative) with the crystallized intelligence composite score (Fig. 9B), and the second latent dimension from 8 parcels had a significant correlation (5 positive and 3 negative) with the crystallized intelligence (Fig. 9C). Example scatter plots of the latent embedding value and the intelligence composite scores are provided in Figure 9D–F.

### 3.5 Low-dimensional latent representations of functional connectivity profiles enable comparisons across area parcels, sessions, subjects, and populations

It is common for neuroimaging analyses to be conducted at the level of areas for biological interpretability (Petersen et al., 2024). However, different atlases exist for defining cortical areas, varying from 100 to 1000 areas across both hemispheres (Arslan et al., 2018; Craddock et al., 2012; Gordon et al., 2016; Schaefer et al., 2018; Shen et al., 2013). In addition to the lack of consensus in area definition, recent work has demonstrated that the optimal definition of areas may vary across individuals (Glasser et al., 2016; Gordon, Laumann, Gilmore, et al., 2017; Kong et al., 2021) and across the lifespan (Han et al., 2018; Myers et al., 2024; Tu et al. 2025). Despite the variation in size and anatomical location of these parcels, we can obtain their FC profiles based on their functional connectivity to each vertex in the cerebral cortex. Subsequently, mapping those FC profiles to the low-dimensional latent space follows the previously described procedures straightforwardly. We mapped the distribution of all areas across all scan sessions in all subjects in two young adult datasets (HCP and MSC) and one baby dataset (BCP). The specific area and network assignments were reproduced in Supplementary Materials (Section A.6).

**Fig. 10.**
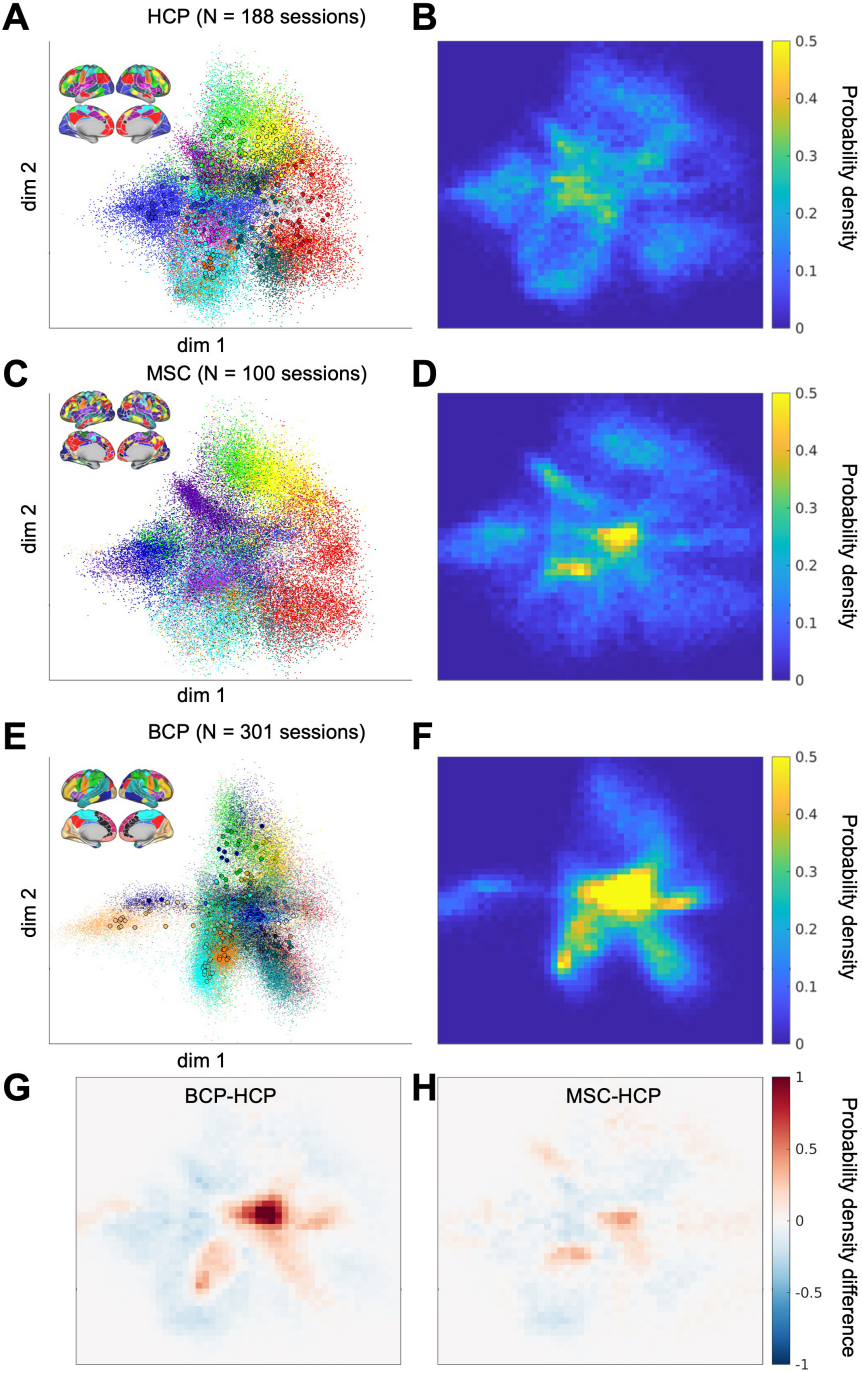
Distribution of functional connectivity profiles from different parcels in different cohorts. (A) FC profiles from 286 area parcels (group-average adult parcellation) in 94 subjects in the HCP dataset with 2 scan sessions each. The network legend for the colors was the same as Figure 4A. (C) FC profiles from 567 to 710 individual-level area parcels in 10 highly-sampled adult individuals with 10 scan sessions each. The area parcels displayed on the brain are from one example subject, with the area parcels and network legend for all subjects in the Supplementary Materials. (E) 326 area parcels (group-average toddler parcellation) in 301 mixed longitudinal and cross-sectional sessions from 178 infant/toddlers aged 8–60 months, with the network legend in the Supplementary Materials. (B, D, F) is the same as (A, C, E) but plotted as a probability density plot. (G) Difference in probability density between BCP and HCP. (H) Difference in probability density between BCP and HCP.

We found that the general compartments occupied by the major network divisions seem consistent across adult and baby datasets (Fig. 10A, C, E). However, the baby dataset (8–60 months) had a different density distribution of points compared to adult datasets (Fig. 10B, D, F). When comparing the baby (BCP) distribution (Fig. 10F) with the young adult (HCP) distribution (Fig. 10B), the BCP distribution was much denser at the center (Fig. 10G), potentially attributable to an overall weaker FC (Fig. 3). On the other hand, the difference in the FC profile embedding distributions in the two young adult datasets (HCP and MSC) (Fig. 10B, C) was much smaller (Fig. 10H). Additionally, the network priors calculated from the average of a group of highly sampled adult individuals for stereotypical appearance of FC in each network (Lynch et al., 2024) also fall within the margins of the expected network distributions from the datasets (Supplementary Fig. A.6).

## 4 Discussion

### 4.1 A low-dimensional latent space facilitates simultaneous comparison of FC profiles across locations and scans

Each point in the latent space represents an FC profile or a spatial connectivity map to all cortical vertices. The distribution of points characterizes the global functional connectivity pattern in each subject/group, which can vary between babies and adults (Fig. 10). Neighboring points in the latent space exhibit coherent spatial patterns and form clusters reminiscent of functional networks (Figs. 3 and 4). On the other hand, the anatomical positions on the brain are uncoupled from the positions in the latent space (Fig. 7). Therefore, similar to other techniques that compare FC independent of the underlying anatomy (Langs et al., 2016), we group nodes (vertices, areas, etc.) together based on their similarity of FC profiles regardless of their anatomical locations. This approach is, thus, ideal for examining large-scale functional networks, which may originate from anatomically isolated compartments.

There are two advantages that our current method has over existing methods of FC embedding (Langs et al., 2016). First, the variational autoencoder is a generative model that offers a straightforward bidirectional mapping between the data space and the latent space, which allows for the visual assessment of example profiles from points in the latent space (Fig. 6). Secondly, our approach facilitates easy projection of out-of-sample data with pre-computed weights, rather than first finding a data-driven embedding space for each subject and then aligning them. This simplifies the comparison across subjects and cohorts with minimal computational effort. To provide a more intuitive example, with a simple function call (see Code Availability), we were able to project the FC profiles from individual areas (approximately 600 per subject) in 100 sessions (10 MSC individual subjects with 10 sessions each) in 523 seconds on a Linux machine equipped with two AMD EPYC 7713 64-core Processors, providing 128 cores and 256 threads, with a base clock speed of 2.56 GHz.

### 4.2 Community detection in a shared latent space naturally establishes correspondence and reduces computational demand

One key pursuit of system neuroscience is to group individual cortical areas into functional networks or communities across states, individuals, and the lifespan (Betzel et al., 2014; Dworetsky et al., 2021; Gordon, Laumann, Adeyemo, & Petersen, 2017; Grayson & Fair, 2017; Kong et al., 2019; Mitra et al., 2017; Muldoon & Bassett, 2016; Puxeddu et al., 2020; Sun et al. 2025; Tagliazucchi et al., 2013; D. Wang et al., 2015) based on their functional connectomes (i.e., area-to-area functional connectivity). A key hurdle for examining those communities is the establishment of correspondence across scan sessions. Prior work has attempted this by detecting communities in individuals and then matching their topographical overlap either through visual inspection (Gordon, Laumann, Gilmore, et al., 2017) or using a Hungarian matching algorithm (Kuhn, 1955) to relabel the networks such that the spatial agreement between corresponding networks is maximized (Langs et al., 2016; Yeo et al., 2014). Alternatively, a multilayer network can be constructed by linking multiple functional connectomes as layers (Bassett et al., 2011; Betzel et al., 2019; Puxeddu et al., 2020), where community detection methods are then applied to the entire multilayer network. However, neither approach can be applied to functional connectomes when the area definitions differ across individuals. At the same time, areas optimized for individuals (Gordon, Laumann, Gilmore, et al., 2017; Kong et al., 2021; Laumann et al., 2015; M. Li et al., 2019) or populations (Han et al., 2018; Myers et al., 2024; Scheinost et al., 2016; Shi et al., 2018; Tu, Myers, et al., 2024; F. Wang et al., 2023) have gained popularity over the years. Moreover, anatomically matched regions across individuals can still vary in their functional properties (Dworetsky et al., 2021; Gordon, Laumann, Adeyemo, & Petersen, 2017; Langs et al., 2016; Mueller et al., 2013).

Clustering in a low-dimensional latent space instead of the original data space can be a computationally efficient (Zhakubayev & Hamerly, 2022) way of solving the correspondence across individuals (Langs et al., 2016; Wen et al., 2024). The FC profiles from different individuals naturally act as a prior for each other to compensate for the short acquisition time in each scan compared to the precision neuroimaging data (Allen et al., 2022; Gordon, Laumann, Gilmore, et al., 2017; Laumann et al., 2015; Lynch et al., 2024; Poldrack et al., 2015).

### 4.3 Challenges and future directions

Our training data represents a small sample with a narrow demographic profile. The training dataset could be expanded to incorporate diverse demographics, acquisition parameters, developmental stages, shorter time windows of dynamic FC patterns, and so on. Additionally, our current study focused on resting-state functional connectivity with a passive viewing or sleep paradigm, but recent studies suggest that a more naturalistic viewing paradigm might better emphasize inter-individual variability (Vanderwal et al., 2017). Future studies could incorporate FC collected in those more naturalistic settings.

To develop a general-purpose model for efficient bidirectional transformation between the spatial FC profiles in the latent space and the vertex space, we chose to directly embed the spatial FC profiles instead of the spatiotemporal maps (Kim et al., 2021, 2023, 2024), even though this overlooks the rich temporal information within fMRI time series. The FC profiles contain individual-specific features (Finn et al., 2015; Gordon, Laumann, Gilmore, et al., 2017; Gratton et al., 2018) and are predictive of cognitive functions (Chen et al., 2022). Being able to run dimensionality reduction directly on the FC profiles from group summaries (e.g., Supplementary Fig. A.6) without access to the raw data can be convenient. Additionally, unlike other factor analysis methods that explore harmonic modes/eigenmodes in the brain based on geometric anatomy (Atasoy et al., 2016, 2018; Pang et al., 2023), the VAE latent space lacks interpretability. Therefore, our model serves as a descriptive/phenomenological model to facilitate exploratory analyses in scientific discovery for functional connectivity analyses only, rather than a mechanistic model to understand the structural basis of functional connectivity.

## 5 Conclusion

The current study demonstrates the application of a variational autoencoder (VAE) to transform functional connectivity profiles from the original vertex space to a low-dimensional latent space. Unlike traditional non-linear dimensionality reduction methods like diffusion map embedding (Langs et al., 2010; Margulies et al., 2016), Laplacian Eigenmap (Haak et al., 2018; Pospelov et al., 2021), isomap (Pospelov et al., 2021), and local linear embedding (Pospelov et al., 2021), the variational autoencoder naturally offers a bidirectional mapping to and from the low-dimensional latent space. This bidirectionality allows the VAE to generate systematically varying FC profiles from samples in the latent space, providing valuable biological intuition. Additionally, by utilizing pre-trained weights from an independent young adult dataset (WU120), the VAE can generate meaningful latent representation without the need for individual-specific latent space computation and post-hoc alignment (Langs et al., 2016; Nenning et al., 2020; Vos de Wael et al., 2020). This latent representation successfully captures the organization of FC profiles into functional networks and highlights the interindividual variability in FC profiles from matching anatomical locations. Our approach offers a robust dimensionality reduction technique for FC profiles across various area parcels, sessions, subjects, and populations, including abstract summary measures such as network priors based on population averages (Lynch et al., 2024). This work could enhance visualization and exploratory analysis for precision functional mapping, providing insights into both individual and group differences in functional connectivity organization across the brain.

## Supplementary Material

Supplementary Material

## Data Availability

Dataset WU120 is available at: https://openneuro.org/datasets/ds000243/versions/00001/file-display/00001 Dataset HCP is available at: https://www.humanconnectome.org/study/hcp-young-adult/document/1200-subjects-data-release Dataset MSC is available at: https://openneuro.org/datasets/ds000224/versions/00001 Dataset BCP is available at: https://nda.nih.gov/edit_collection.html?id=2848 The Pytorch implementation for the beta-VAE can be found at https://github.com/cindyhfls/Tu-2025-VAE_FC_embedding, which was adapted from: https://github.com/libilab/rsfMRI-VAE. Pre-trained model weights can be found at https://huggingface.co/cindyhfls/fcMRI-VAE. Visualization is conducted through Connectome Workbench (https://www.humanconnectome.org/software/connectome-workbench) and custom MATLAB scripts (https://github.com/cindyhfls/MATLAB_BrainParcelVisualizationFunctions).
