# Supplementary material for: Deep learning-based embedding of functional connectivity profiles for precision functional mapping
Source: Imaging Neurosci (Camb). 2025 Sep 3;3:IMAG.a.129. doi: 10.1162/IMAG.a.129 (PMC12409741; doi:10.1162/IMAG.a.129)
Supplement: Supplementary Material [file IMAG.a.129_supp.pdf]

Supplementary Materials for "Deep-learning based Embedding of Functional Connectivity Profiles for Precision Functional Mapping"

Jiaxin Cindy Tu[1], Jung-Hoon Kim[2], Chenyan Lu[1], Patrick Luckett[3], Babatunde Adeyemo[1], Joshua S. Shimony[1], Jed T. Elison[4,5], Adam T. Eggebrecht[1], Muriah D. Wheelock[1]

[1]Mallinckrodt Institute of Radiology, Washington University in St. Louis, [2]Developing Brain Institute, Children's National Hospital, [3]Department of Neurosurgery, Washington University, [4]Institute of Child Development, University of Minnesota, [5]Masonic Institute for the Developing Brain, University of Minnesota.

Section A. Neuroimaging Datasets

A.1 Washington University 120 (WU120) Data Acquisition and Processing Details

This dataset has been extensively detailed in prior descriptions (Power et al., 2014). In summary, data were obtained from 120 healthy young adults, with relaxed eyes-open fixation (60 females, average age = 25 years, age range = 19–32 years). Participants were right-handed, native English speakers recruited from the Washington University community. Screening via self-report questionnaire ensured no history of neurological or psychiatric diagnosis, nor head injuries resulting in more than 5 minutes of unconsciousness. All participants provided informed consent, and the study was approved by the Washington University School of Medicine Human Studies Committee and Institutional Review Board.

Structural and functional MRI data were obtained with a Siemens MAGNETOM Trio Tim 3.0-T Scanner and a Siemens 12-channel Head Matrix Coil. Structural imaging included a T1-weighted sagittal magnetization-prepared rapid acquisition gradient-echo (MP-RAGE) structural image was obtained [time echo (TE) = 3.08 ms, time repetition, TR (partition) = 2.4 s, time to inversion (TI) = 1000 ms, flip angle = 8°, 176 slices with 1 × 1 × 1 mm voxels]. Functional scan slices were aligned parallel to the anterior commissure–posterior commissure plane of the MP-RAGE and centered on the brain using an auto-align pulse sequence protocol available in Siemens software. This alignment corresponds to the Talairach atlas (Talairach & Tournoux, 1988).

For the fMRI data acquisition, subjects were instructed to relax while maintaining fixation on a black crosshair against a white background. Functional imaging used a BOLD contrast-sensitive gradient-echo echo-planar imaging (EPI) sequence (TE = 27 ms, flip angle = 90°, in-plane resolution = 4 × 4 mm). Full-brain EPI volumes (MR frames) of 32 contiguous, 4-mm-thick axial slices were obtained every 2.5 seconds. Additionally, a T2-weighted turbo spin-echo structural image (TE = 84 ms, TR = 6.8 s, 32 slices with 1 × 1 × 4 mm voxels) in the same anatomical planes as the BOLD images was also captured to augment atlas alignment. The fMRI acquisition used Anterior→Posterior (AP) phase encoding. The number of volumes collected per subject ranged from 184 to 724, with an average of 336 frames (14.0 min).

Functional images were first processed to reduce artifacts including (1) correction of odd versus even slice intensity differences due to interleaved acquisition without gaps, (2) head movement correction within and across runs, and (3) across-run intensity normalization to a whole-brain mode value of 1000 (Miezin et al., 2000). Each individual's functional data was transformed into an atlas space using the MP-RAGE scan and resampled to an isotropic 3-mm atlas space

(Talairach and Tournoux, 1988), using a single cubic spline interpolation (Lancaster et al., 1995).

Additional preprocessing mitigated high-motion frame effects in two iterations. The first iteration included: (1) demeaning and detrending, (2) multiple regression including whole-brain, ventricular cerebrospinal fluid (CSF), and white matter signals, and motion regressors derived by Volterra expansion, and (3) a band-pass filter (0.009 Hz < f < 0.08 Hz). Temporal masks were created in this iteration to flag motion-contaminated frames, identified using framewise displacement (FD), calculated as the squared sum of the motion vectors (Power et al., 2012). Volumes exceeding FD > 0.2 mm and segments of fewer than 5 contiguous volumes were flagged for removal.

The data were then reprocessed in a second iteration, incorporating the temporal masks described above. This reprocessing was identical to the initial processing stream but ignored censored data. Data were interpolated across censored frames using least squares spectral estimation (Power et al., 2014) of the values at censored frames so that continuous data could be passed through the band-pass filter (0.009 Hz < f < 0.08 Hz) without contaminating frames near high-motion frames. *Censored frames were ultimately ignored when calculating the functional connectivity profiles.*

Individual surfaces were generated from the structural images, and functional data were sampled to surface space (Glasser et al., 2013). Following volumetric registration, left and right hemisphere anatomical surfaces were created from each subject's MP-RAGE image using FreeSurfer's recon-all processing pipeline (v5.0)(Fischl, 2012). This involved brain extraction, segmentation, white matter and pial surface generation, surface inflation to a sphere, and spherical registration of the subject's "native" surface to the fsaverage surface. The fsaverage-registered surfaces were then aligned and resampled to a resolution of 164000 vertices using Caret tools (Van Essen et al., 2001) and down-sampled to a 32492-vertex surface (32k-fs_LR). Functional BOLD volumes were sampled to each subject's individual "native" midthickness surface (generated as the average of the white and pial surfaces) using the ribbon-constrained sampling procedure available in Connectome Workbench (v0.84) and then deformed and resampled from the individual's "native" surface to the 32k-fs_LR surface. The final time series was smoothed along the 32k-fs_LR surface using a Gaussian smoothing kernel (σ = 2.55 mm).

A.2 Human Connectome Project (HCP) Data Acquisition and Processing Details

Data for the Human Connectome Project (Young Adult) was collected at Washington University in St. Louis and the University of Minnesota. The participants, healthy adults aged between 22 to 35 years, underwent high-resolution T1-weighted (MP-RAGE, TR = 2.4s, voxel size = 0.7×0.7×0.7mm) and BOLD contrast-sensitive imaging (gradient echo EPI, multiband factor 8, TR = 0.72s, voxels = 2×2×2 mm) using a custom Siemens SKYRA 3.0T MRI scanner equipped with a custom 32-channel Head Matrix Coil. Sequences with both left-to-right (LR) and right-to-left (RL) phase encoding were employed, with each participant completing a single run in each direction over two consecutive days, resulting in four runs in total, two for Rest1 and another two for Rest2 (Van Essen, Ugurbil, et al., 2012).

Functional data processing first followed the HCP minimally preprocessing pipeline (Glasser et al., 2013), which included the additional use of a field map for distortion correction (Cusack et al., 2003; Jezzard & Balaban, 1995) and otherwise similar steps as mentioned above. Then, additional preprocessing of the resting-state BOLD volume data was applied to remove non-neuronal sources of artifacts similar to the WU120 data, with the only differences in and an added low-pass filter at 0.1 Hz applied on the movement parameters before calculating FD to

mitigate high-frequency respiration artifacts on FD estimates (Gratton, Dworetsky, et al., 2020). Frames with FD greater than 0.04 mm were flagged as “high-motion” based on the noise floor in FD traces. Instead of processing in two iterations as mentioned above for the WU120 data, the temporal mask excluding high-motion frames based on FD was used in the calculation of regression coefficients and linear interpolation before the application of bandpass filtering. *Censored frames were ultimately ignored when calculating the functional connectivity profiles.*

Following that, the preprocessed BOLD volumes were sampled to each subject's individual “native” midthickness surface (generated as the average of the white and pial surfaces) using the ribbon-constrained sampling procedure available in Connectome Workbench and then deformed and resampled from the individual's “native” surface to the 32k-fs_LR surface. The final BOLD time series was minimally smoothed with a Gaussian kernel (FWHM = 2mm, σ = 0.85).

A.3 Midnight Scan Club (MSC) Acquisition and Processing Details

The acquisition and processing of this dataset have been mentioned in elaborate detail in a prior study (Gordon, Laumann, Gilmore, et al., 2017). Data were collected from ten healthy, right-handed, young adult subjects (5 females; age: 24-34) recruited from the Washington University community. The study was approved by the Washington University School of Medicine Human Studies Committee and Institutional Review Board.

Imaging for each subject was performed on a Siemens TRIO 3T MRI scanner over the course of 12 sessions conducted on separate days, each beginning at midnight. Structural MRI was conducted across two separate days. In total, four T1-weighted images (sagittal, 224 slices, 0.8 mm isotropic resolution, TE = 3.74 ms, TR = 2400 ms, TI = 1000 ms, flip angle = 8 degrees), four T2-weighted images (sagittal, 224 slices, 0.8 mm isotropic resolution, TE = 479 ms, TR = 3200 ms) were obtained for each subject. On ten subsequent days, each subject underwent 1.5 hr of functional MRI scanning beginning at midnight. In each session, we first collected thirty contiguous minutes of resting state fMRI data, in which subjects visually fixated on a white crosshair presented against a black background. Each subject was then scanned during the performance of three separate tasks: motor (2 runs per session, 7.8 min combined), incidental memory (3 runs per session, 13.1 min combined), and mixed design (2 runs per session, 14.2 min combined). Across all sessions, each subject was scanned for 300 total minutes during the resting state and approximately 350 total minutes during task performance. All functional imaging was performed using a gradient-echo EPI sequence (TR = 2.2 s, TE = 27 ms, flip angle = 90°, voxel size = 4 mm x 4 mm x 4 mm, 36 slices). In each session, one gradient echo field map sequence was acquired with the same prescription as the functional images.

Following that, the preprocessed BOLD volumes were sampled to each subject's individual “native” midthickness surface (generated as the average of the white and pial surfaces) using the ribbon-constrained sampling procedure available in Connectome Workbench and then deformed and resampled from the individual's “native” surface to the 32k-fs_LR surface. The final BOLD time series was smoothed with a Gaussian kernel (FWHM = 6mm, σ = 2.55).

A.4 Baby Connectome Project Acquisition and Processing Details

Details about the acquisition and processing of this dataset have been previously reported (Tu, Wang, et al., 2024). Full-term (gestational age of 37-42 weeks) infants free of any major pregnancy and delivery complications were recruited as part of the Baby Connectome Project (Howell et al., 2019). All procedures were approved by the University of North Carolina at

Chapel Hill and the University of Minnesota Institutional Review Boards. Informed consent was obtained from the parents of all participants. In the final cohort used following fMRI data quality control, we retained 301 fMRI sessions from 178 individuals acquired during natural sleep. All MRI images were acquired on a Siemens 3T Prisma scanner with a 32-channel head coil at the University of Minnesota and the University of North Carolina at Chapel Hill during natural sleep without the use of sedating medications. T1-weighted (TR=2400 ms, TE=2.24 ms, 0.8 mm isotropic; flip angle = 8°), T2-weighted images (TR=3200 ms, TE=564 ms, 0.8 mm isotropic), spin echo field maps (SEFM) (TR=8000 ms, TE=66 ms, 2 mm isotropic, MB=1), and fMRI data (TR=800 ms, TE=37 ms, 2 mm isotropic, MB=8) were collected. A mixture of Anterior→Posterior (AP) and Posterior→Anterior (PA) phase encoding directions was used for fMRI acquisition in each session, but they were concatenated into one time series. A subset of data had a 720-ms TR.

Data processing used the DCAN-Labs infant-abcd-bids-pipeline (v0.0.22), which largely follows the HCP processing(Glasser et al., 2013) and the steps described previously for the ABCD dataset (Feczko et al., 2021). The processing steps were similar to the adult data except for the use of infant-specific MNI templates to better register the structural data. In addition, segmentation of the brain structures was conducted with Joint Label Fusion (JLF). The toddler-specific brain mask and segmentation were substituted to the Freesurfer (Fischl, 2012) pipeline to refine the white matter segmentation and guide the FreeSurfer surface delineation for each scan session of each subject. The native surface data were then deformed to the 32k fs_LR template via a spherical registration.

For functional data processing, a scout image (frame 16 in each run) was selected from the fMRI time series. The scout was distortion-corrected via spin-echo field maps, served as the reference for motion correction via rigid-body realignment (Feczko et al., 2021), and was registered to the native T1. Across-run intensity normalization to a whole-brain mode value of 10,000 was then performed. These steps were combined in a single resampling with the MNI template transformation from the previous step, such that all fMRI frames were registered to the infant MNI template. Manual inspection of the image quality of structural and functional data was conducted to exclude sessions with bad data quality. fMRI BOLD volumes were sampled to native surfaces using a ribbon-constrained sampling procedure available in Connectome Workbench and then deformed and resampled from the individual's “native” surface to the 32k-fs_LR surface. Additional steps to mitigate the non-neuronal sources of artifact including demean/detrend, nuisance regression and bandpass filtering was conducted on the surface data similar to the adults with a few minor differences: 1) gray matter signal based on the 91k grayordinates in the 32k-fs_LR surface substituted the whole-brain signal in the nuisance regression, 2) a respiratory notch filter (0.28-0.48 Hz) was applied to the motion parameter estimates before FD calculation to minimize the inflation of FD values by the perturbation of magnetic field from subject respiration in fast TR scans (Fair, 2020; Kaplan et al., 2022). The data were originally minimally spatially smoothed with a geodesic 2D Gaussian kernel (σ = 0.85 mm). A further smoothing with a geodesic 2D Gaussian kernel (σ = 2.40 mm) was applied to give a final effective smoothing of σ = 2.55 mm.

A.5 Parcellation Atlases Used to Define Areas and Functional Networks in Each Dataset

The area parcellation atlases used for MSC data (Supplementary Figure A.5A) and BCP data (Supplementary Figure A.5B) with their network assignments were reproduced below from the original publications.

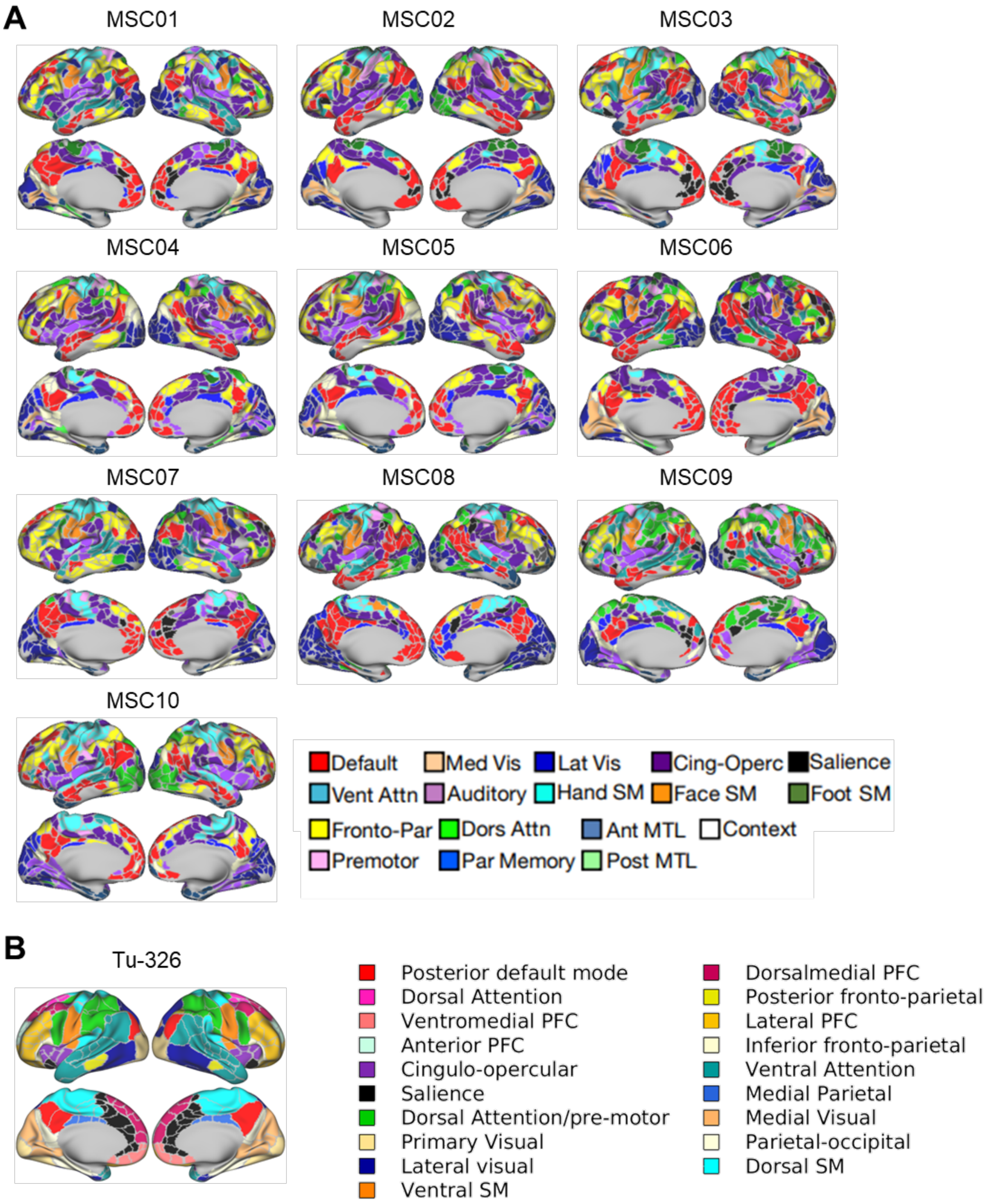

Supplementary Figure A.5 Parcellations used for defining areas and networks for the MSC and BCP datasets. A) Subject-specific parcellation and putative network identity of each parcel for each MSC subject. Reproduced from Gordon, E. M., Laumann, T. O., Gilmore, A. W., Newbold, D. J., Greene, D. J., Berg, J. J., ... & Dosenbach, N. U. (2017). Precision functional mapping of individual human brains. Neuron, 95(4), 791-807. Copyright 2017 by Elsevier Inc. Default = Default mode. Med Vis = Medial Visual. Lat Vis = Lateral Visual. Vent Attn = Ventral Attention. SM = Somatomotor. Fronto-Par = Fronto-parietal. Dors Attn = Dorsal Attention. Ant

MTL = anterior medial temporal lobe. Posterior MTL = posterior medial temporal lobe. Par Memory = Parietal memory. B) Group-average toddler parcellation.

A.6 Functional Network Priors from Highly-sampled Adult Individuals

We reproduced the functional network priors figure calculated from 45 adult individuals (Lynch et al., 2024) from the original publication and indexed them to the locations on the 2D latent space.

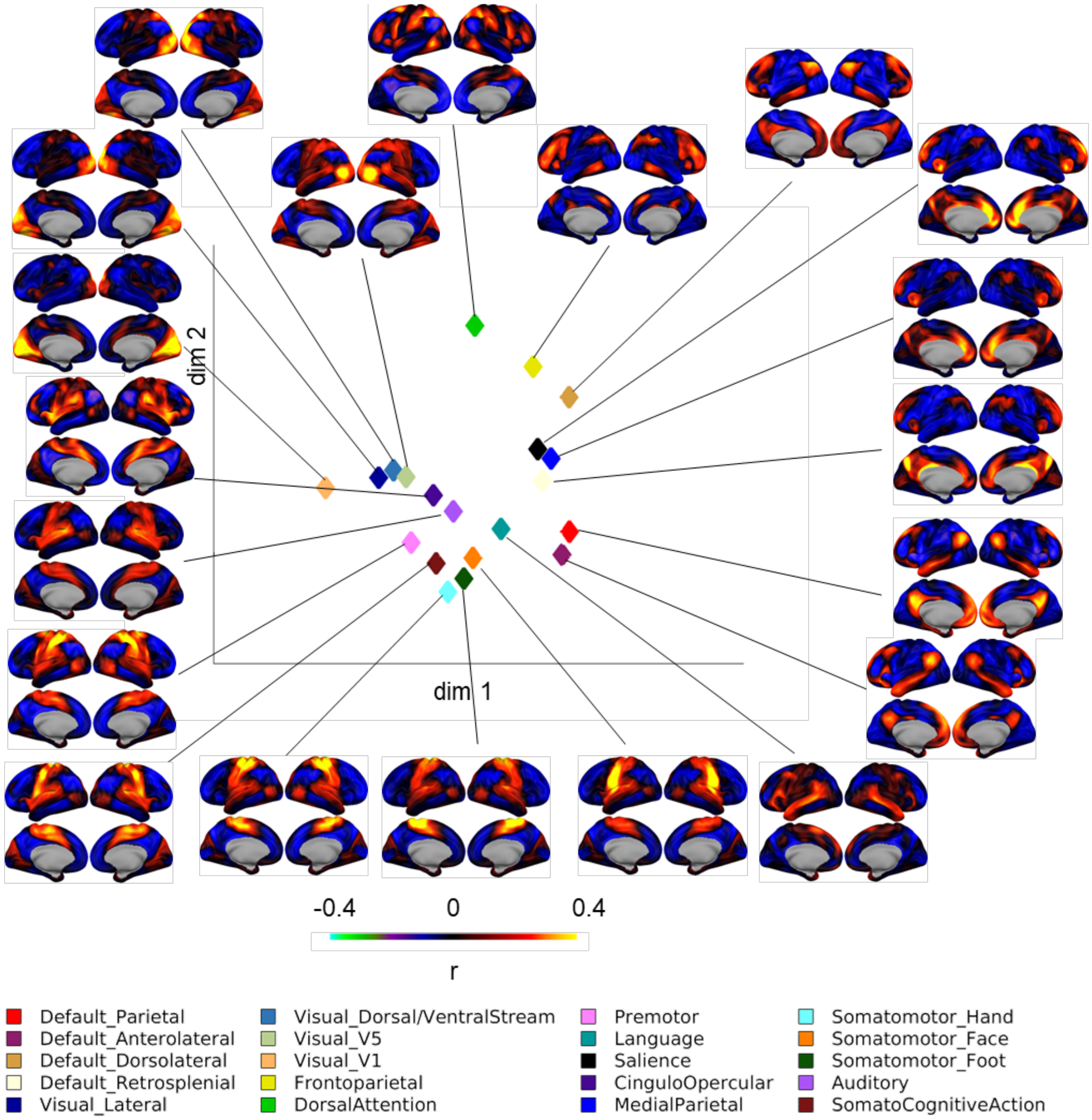

Supplementary Figure A.6 Functional priors and their location on the 2-D latent space. The functional network priors were reproduced from data shared by Lynch, C.J., Elbau, I.G., Ng, T. et al. Frontostriatal salience network expansion in individuals with depression. Nature 633, 624–633 (2024).

Section B. Model Training Details

B.1 Model Architecture

The variational autoencoder consists of a total of 12 layers (Figure B.1). In the encoder network, the size of the output image of each layer (from left to right) is 96x96x64 (32 channels per hemisphere), 48x48x128, 24x24x128, 12x12x256, and 6x6x256; for the decoder network, 6x6x256, 12x12x256, 24x24x128, 48x48x128, and 96x96x64 (32 channels per image), from left to right. The dimension of latent variables is 256. The convolution operations are defined as 1: convolution (kernel size=8, stride=2, padding=3) with rectified nonlinearity, 2-5: convolution (kernel size=4, stride=2, padding=1) with rectified nonlinearity, 6: fully-connected layer with re-parametrization, 7: fully-connected layer with rectified nonlinearity, 8-11: transposed convolution (kernel size=4, stride=2, padding=1) with rectified nonlinearity, 12: transposed convolution (kernel size=8, stride=2, padding=3).

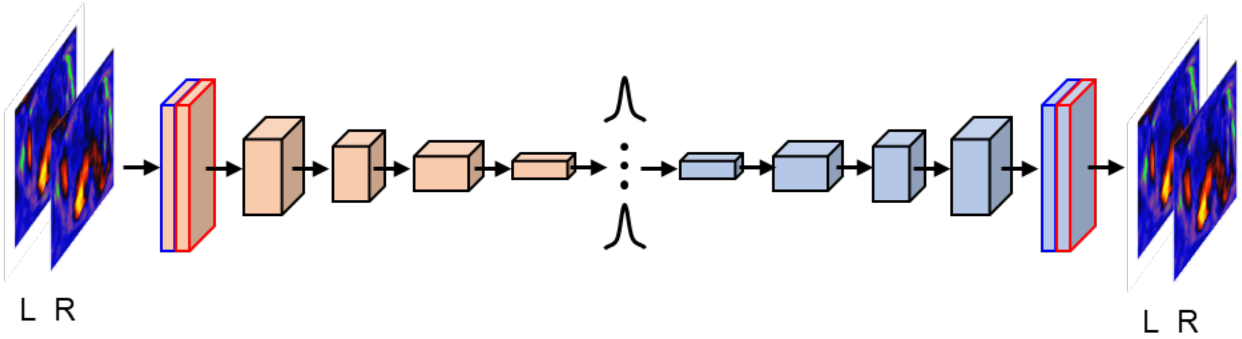

Supplementary Figure B.1 *Model Architecture*. Adapted from Kim, J., Zhang, Y., Han, K., Wen, Z., Choi, M., & Liu, Z. (2021). Representation learning of resting state fMRI with variational autoencoder. NeuroImage, 241, 118423. Copyright 2021 by Elsevier Inc.

B.2 Reconstruction Performance

We demonstrate that the original FC profiles from vertices, areas, functional networks, or even functional network priors can be embedded and reconstructed using the VAE encoder and decoder, as shown in a test subject from the WU120 dataset (Supplementary Figure B.2). It is important to note that perfect reconstruction may not be ideal, as the original data could contain noise. The reconstructed data might therefore represent a denoised version of the original data.

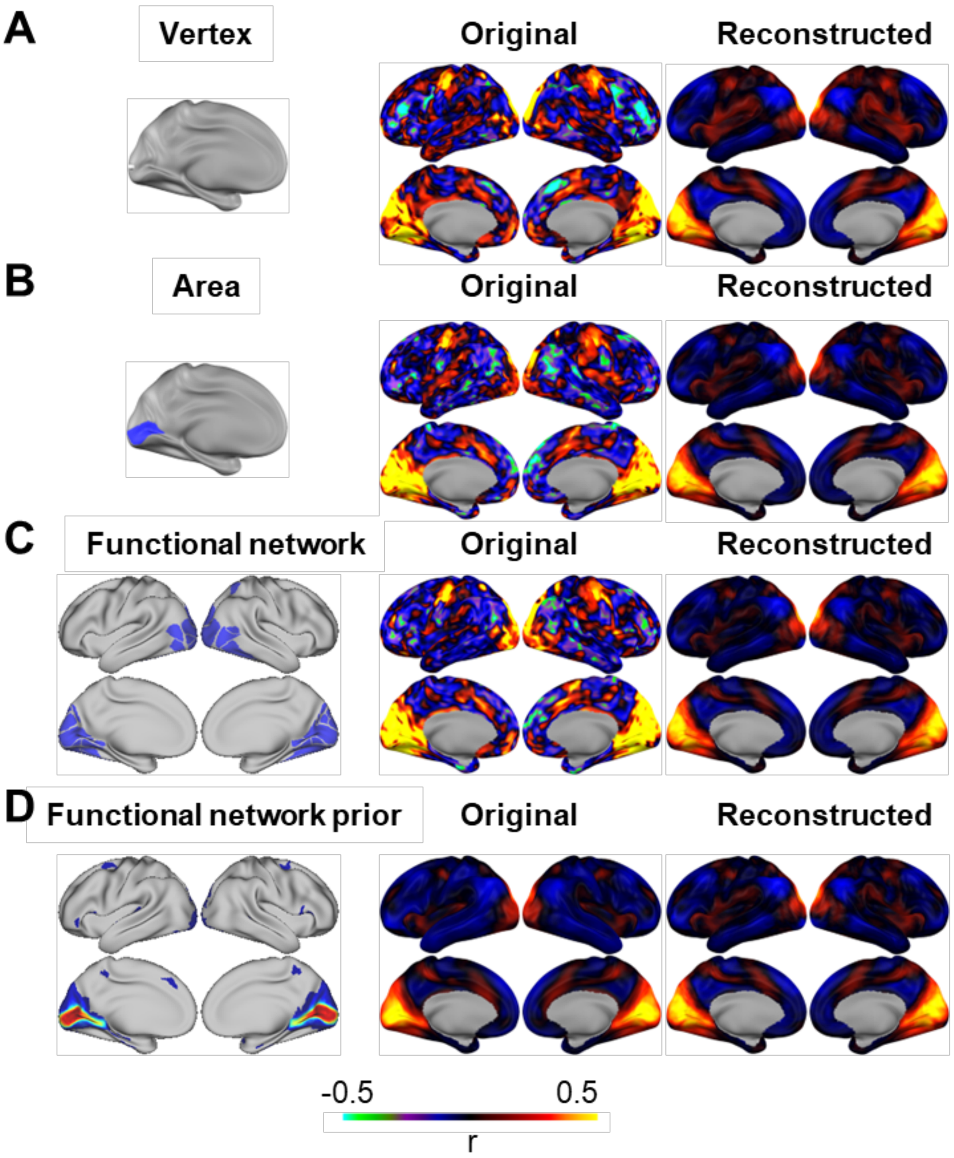

Supplementary Figure B.2 *Original and reconstructed FC profiles from the beta-VAE model.* A) from a vertex, B) from an area, C) from a functional network, and D) from group-averaged functional network connectivity (a.k.a. functional network priors). A-C is from one example subject in WU120. D is reproduced from data shared by Lynch, C.J., Elbau, I.G., Ng, T. et al. Frontostriatal salience network expansion in individuals with depression. Nature 633, 624–633 (2024).

B.3 Hyperparameter Tuning

Our goal was to learn a regularized latent space that would generalize well to unseen data without significantly compromising reconstruction accuracy. To achieve this, we evaluated the reconstruction loss and KL divergence in models with β values ranging from 1 to 250. We found that β = 20 offered a good balance between reconstruction performance and KL divergence on the validation data, as shown in Supplementary Figure B.3.

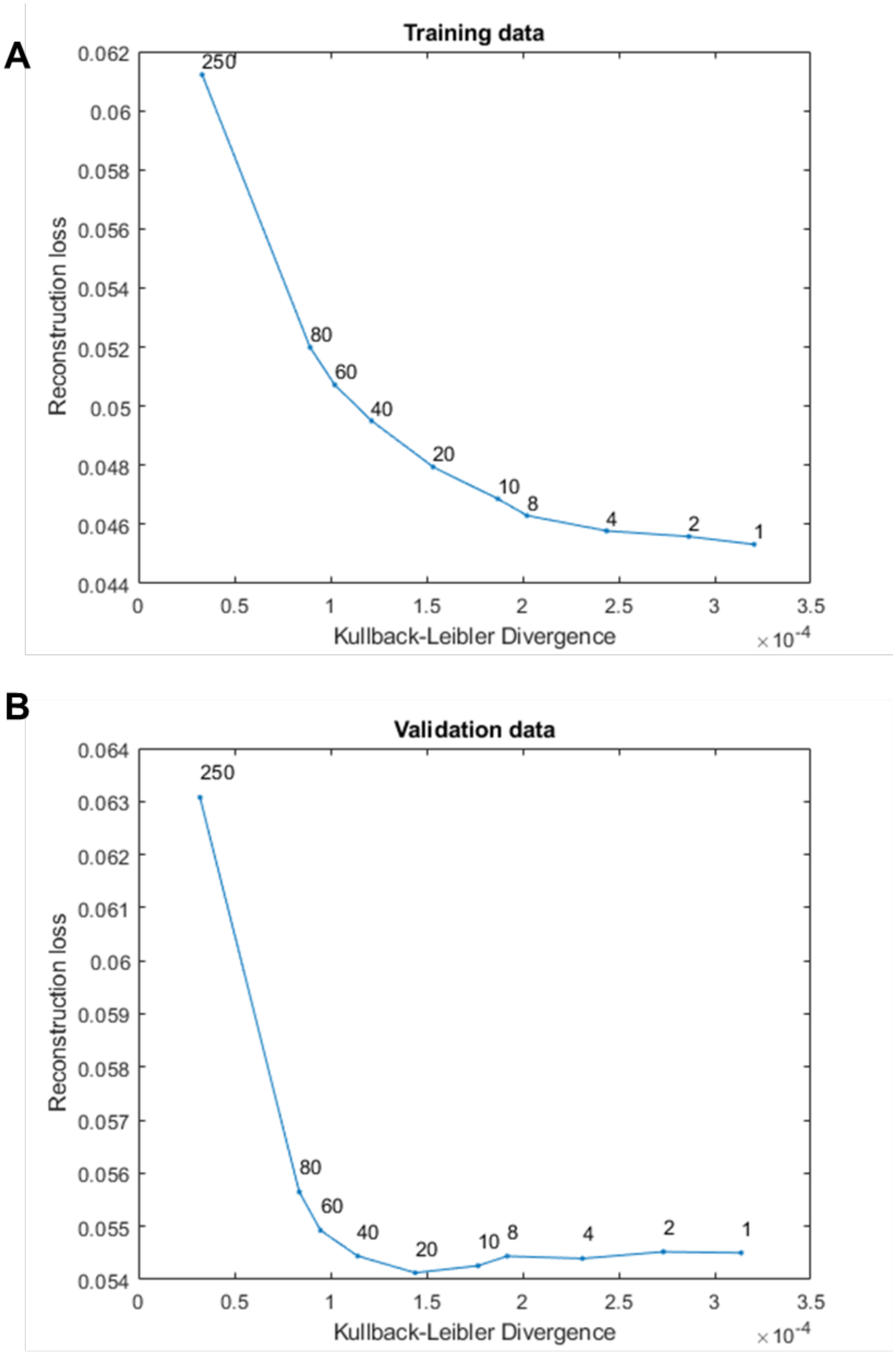

Supplementary Figure B.3 *Hyperparameter tuning.* A) Reconstruction loss and Kullback-Leibler divergence for different beta values in the training data. B) Reconstruction loss and Kullback-Leibler divergence for different beta values in the validation data.

Section C. VAE Latent Space with Different Numbers of Dimensions.

The mean silhouette index (SI) in the 2D latent space was significantly above zero for the average FC profile embedding from 286 areas within 12 networks across 94 HCP subjects (Figure 4). However, it was lower than the SI in the original vertex space using the correlational distance. To investigate whether certain networks would be better segregated with more latent dimensions, we replicated Figure 4 using latent dimensions of 3, 4, 8, 16, and 32. We found that both the mean SI and the correlation between distances in the latent space and vertex space increased sharply from 2 to 4 dimensions (Supplementary Figure C.1-1). The values peaked around 8 dimensions before decreasing with further increases in latent dimensions. Some networks, such as the parietal memory and fronto-parietal networks, as well as the somatomotor hand and foot networks, become more segregated at higher dimensions (Supplementary Figure C.1-2). Therefore, the 4-dimensional VAE latent space could be particularly useful for visualizing these finer separations that were not well captured in 2 dimensions (Figure 4).

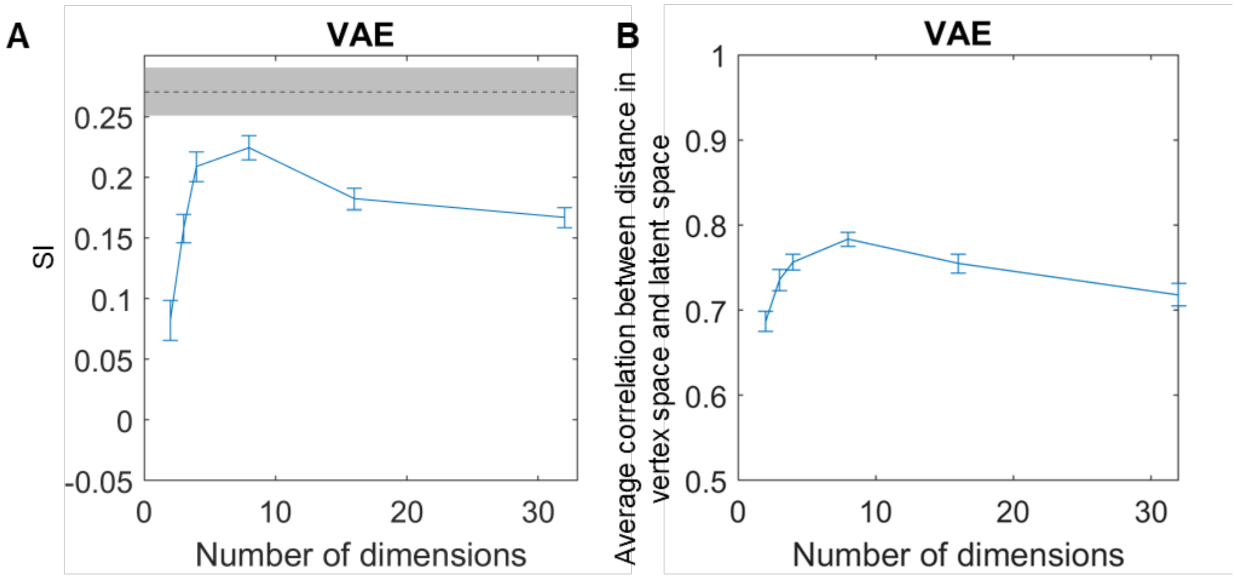

Supplementary Figure C.1-1 *VAE Latent Space with Different Numbers of Dimensions.* A) Mean SI across all areas in the 12 networks using Euclidean distance between the embeddings in the latent space. B) Average correlation between the (correlational) distance in the vertex space and the (Euclidean) distance in the latent space. The line plot shows the metric calculated with the whole 94-subject sample, and the error bars show the 95% bootstrapped confidence interval.

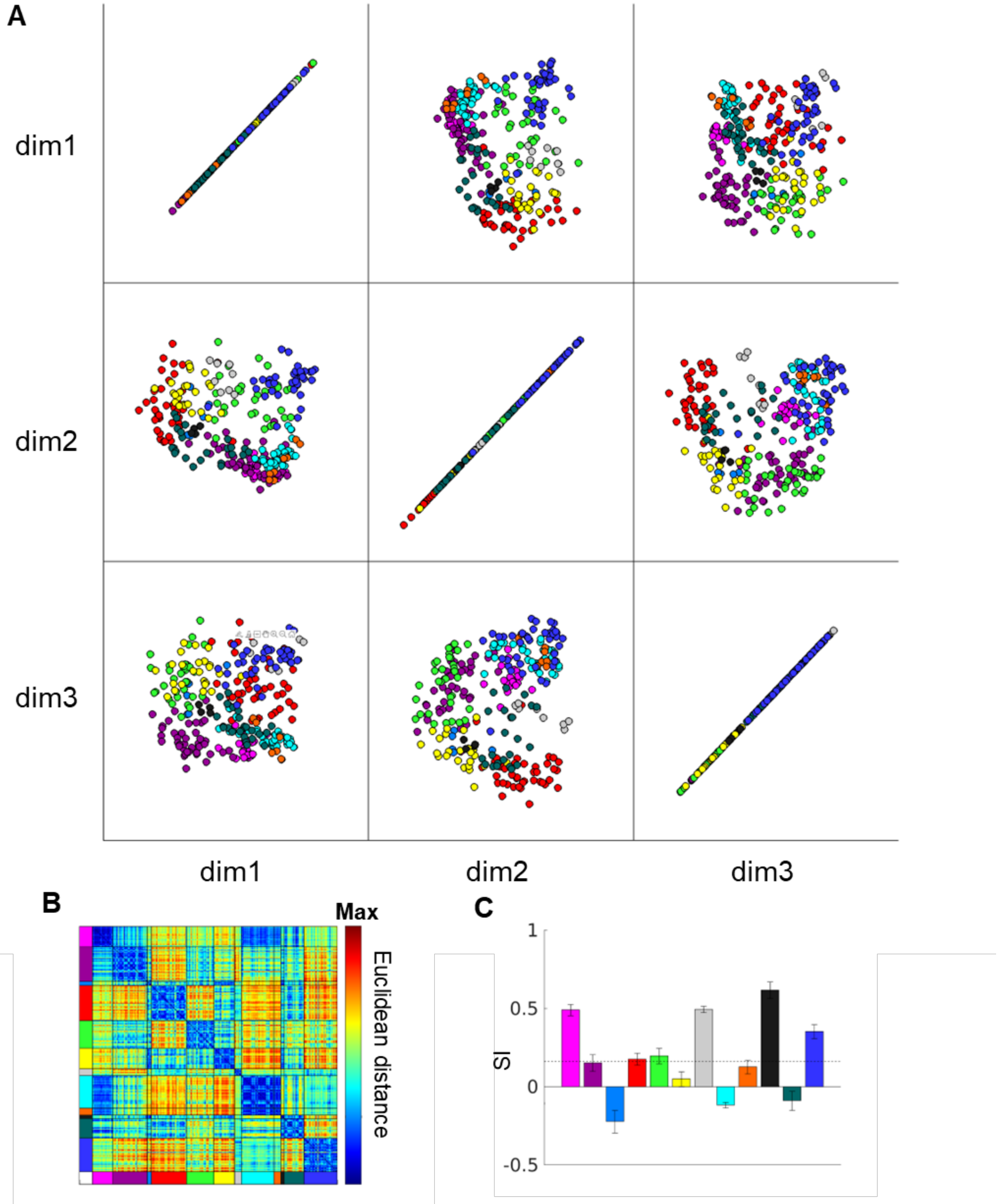

Supplementary Figure C.1-2 Separation of functional connectivity profiles by functional networks in the average of 94 HCP subjects (Rest1) - 3 dimensions. A) The FC profile latent embeddings with three dimensions in VAE. Each circle represents each area parcel's mean functional connectivity profile across Rest1 sessions of 94 subjects. B) The mean

Euclidean distance between the latent representations of the average across 94 subjects. C) The mean silhouette index for each functional network based on the Euclidean distance in B.

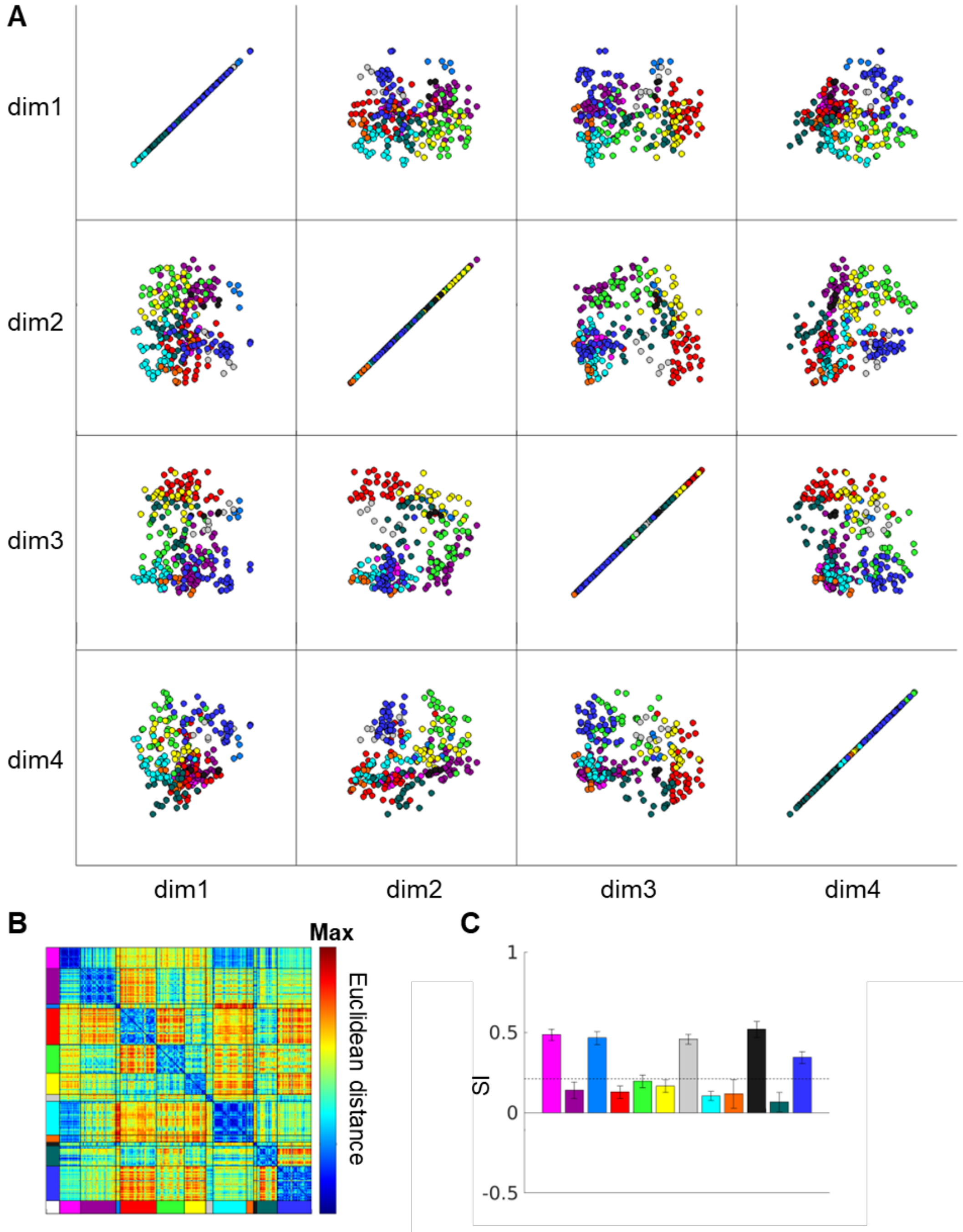

Supplementary Figure C.1-3 Separation of functional connectivity profiles by functional networks in the average of 94 HCP subjects (Rest1) - 4 dimensions. A) The FC profile latent embeddings with three dimensions in VAE. Each circle represents each area parcel's mean functional connectivity profile across Rest1 sessions of 94 subjects. B) The mean

Euclidean distance between the latent representations of the average across 94 subjects. C) The mean silhouette index for each functional network based on the Euclidean distance in B.

Section D. Comparison to Alternative Autoencoder-based and Linear Dimensionality Reduction Methods

D.1 Alternative Autoencoder-based and Linear Dimensionality Reduction Methods

We selected the beta-VAE for its previously demonstrated ability to disentangle interpretable factors from images and to learn a continuous latent space for generating new data (Higgins et al., 2017). For the sake of completeness, we also tested the dimensionality of the FC profiles using a conventional autoencoder (Supplementary Figure D.1B), which learns a deterministic latent representation instead of a distribution, and an adversarial autoencoder (Makhzani et al., 2016) (Supplementary Figure D.1C), which employs a generative adversarial network (Goodfellow et al., 2014) based architecture to regularize the latent space.

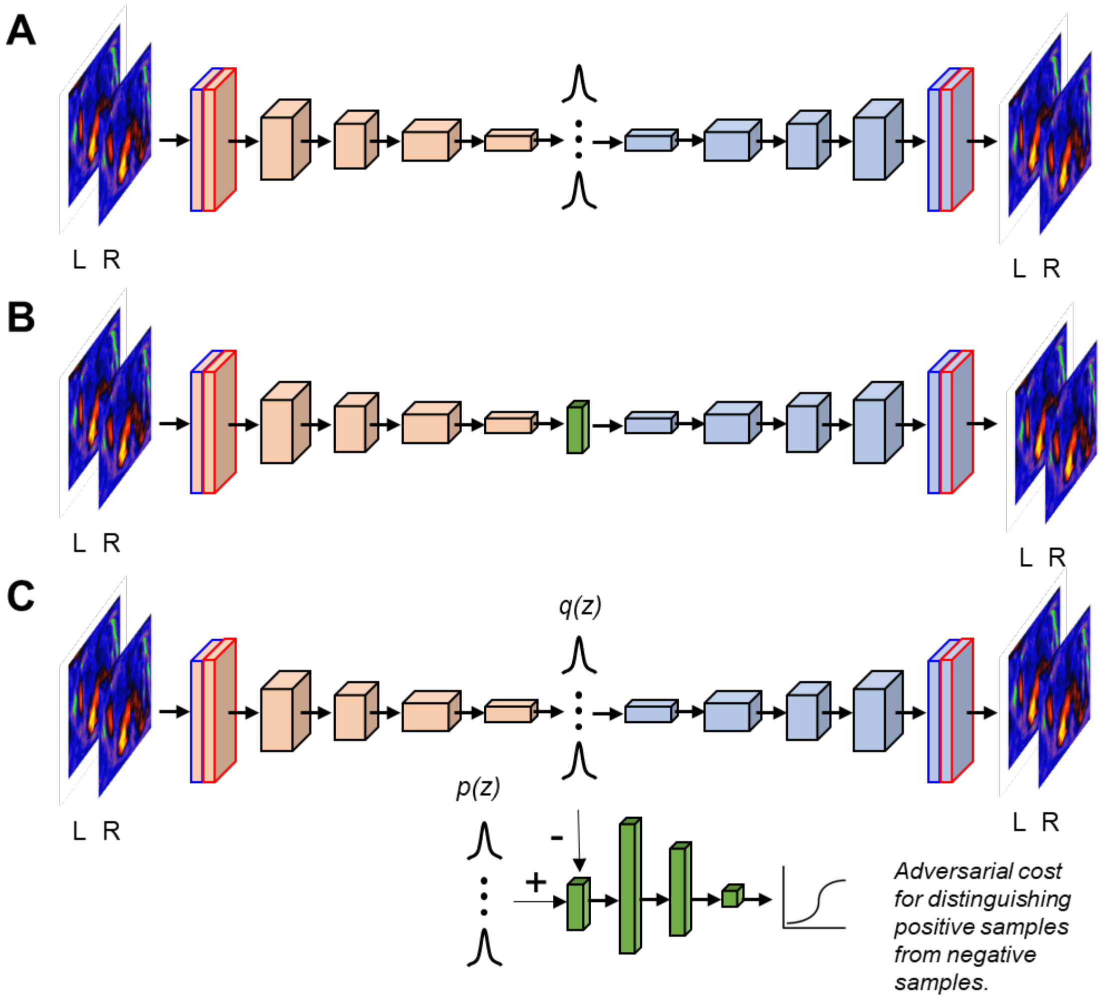

Figure D.1 Autoencoder variants. A) Variational autoencoder. B) Autoencoder. C) Adversarial autoencoder. Blue and red boxes stand for the input images from the left and right hemispheres, respectively. Adapted from Kim, J., Zhang, Y., Han, K., Wen, Z., Choi, M., \& Liu, Z. (2021). Representation learning of resting state fMRI with variational autoencoder. NeuroImage, 241,

118423. Copyright 2021 by Elsevier Inc. and Makhzani, A., Shlens, J., Jaitly, N., Goodfellow, I., Frey, B., ICLR 2016. Adversarial Autoencoders.

We chose principal component analysis (PCA) and independent component analysis (ICA) as alternative linear dimensionality reduction methods. PCA employs Singular Value Decomposition of the data, retaining only the most significant singular vectors to project the data into a lower-dimensional space. ICA aims to decompose the data into a set of independent spatial maps. For memory management purposes, we performed PCA using incremental PCA (IPCA) from the Scikit-learn package (v1.3.2) in Python 3.8 (Golub & Loan, 2013; Ross et al., 2008). IPCA builds a low-rank approximation of the input data while using a constant memory amount regardless of the number of samples. ICA was then performed on the reduced data with the first 100 principal components using FastICA in Scikit-learn. To maintain fairness across algorithms, we provided the geometrically reformatted FC profile (from the 192 x 192 grid) to PCA and ICA instead of the original FC profile.

D.2 Single Latent Traversal

As before, we varied the magnitude of one latent dimension while keeping the other latent dimension fixed at zero. The most prominent divisions between sensorimotor networks and association networks (Margulies et al., 2016; Sydnor et al., 2021), as well as between task-positive to task-negative networks (Buckner et al., 2008; M. D. Fox et al., 2005; Raichle, 2015), remained evident in most of the autoencoder-based and linear dimensionality reduction methods. However, only the VAE models, both with regularization ($\beta = 20$) and without regularization ($\beta = 1$), were capable of demonstrating the subtle transitions across the somatomotor networks.

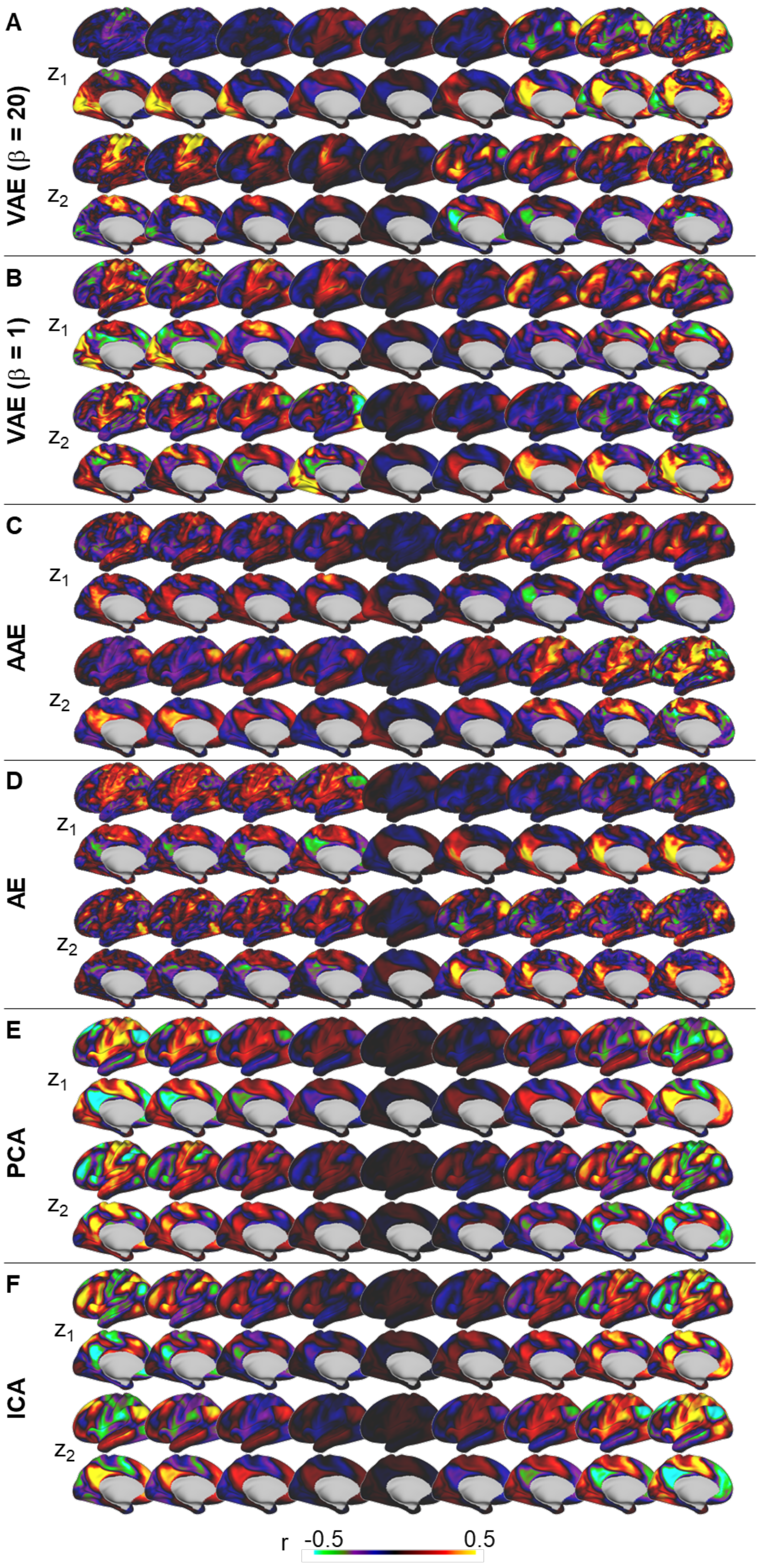

A
VAE (β = 20)
z1
z2
B
VAE (β = 1)
z1
z2
C
AAE
z1
z2
D
AE
z1
z2
E
PCA
z1
z2
F
ICA
z1
z2
r -0.5 0.5

Supplementary Figure D.2. **Single latent traversal.** The reconstructed FC profiles from latent values of one dimension varied in equal steps from one end to the other end, and the other dimension was fixed to 0. A) VAE (β = 20), B) VAE (β = 1), C) AAE, D) AE, E) PCA, F) ICA. VAE = variational autoencoder. AAE = adversarial autoencoder. AE = autoencoder. PCA = principal component analysis. ICA = independent component analysis.

D.3 Functional Networks Organization in the Latent Space

At two dimensions, the average SI for VAE (β = 20) best separated the functional networks with a mean SI of 0.086 (95% CI: [0.066, 0.099]), higher than the VAE (β = 1) (0.044, 95% CI: [0.015, 0.063]), AAE (0.035, 95% CI: [0.006,0.054]), AE (0.014, 95% CI: [-0.018,0.030]), PCA (7×10^{-4}, 95% CI: [-0.016, 0.009]) and ICA (-0.010, 95% CI: [-0.028, 0.001]) (Supplementary Figure D.3-1 & D.3-2).

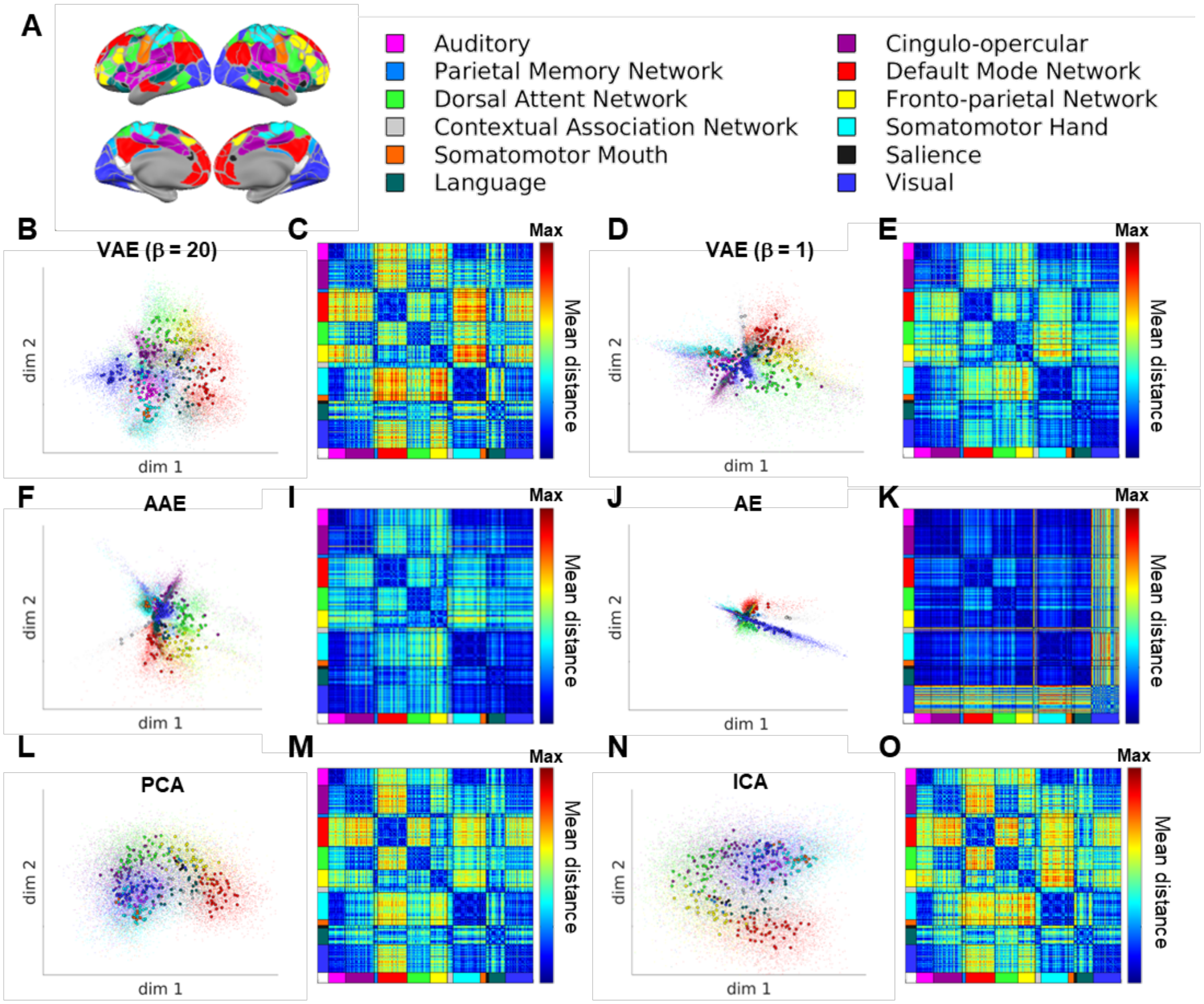

Supplementary Figure D.3-1. **Separation of functional connectivity profiles by functional networks in the average of 94 HCP subjects (Rest1).** A) Gordon network assignments for 286 area parcels. B) The FC profile latent representations with two dimensions in VAE. Each circle represents each area parcel's mean functional connectivity profile across Rest1 sessions of 94 subjects. Each dot represents an area parcel from one subject. C) The mean Euclidean distance between the latent representations of the average across 94 subjects. D-O) Same as B-C for alternative dimensionality reduction methods. The individual dots were displayed here to

demonstrate the intersubject variability in FC profile embeddings, but the distance matrices (e.g., panel B) were generated using the average FC profile embeddings across subjects (circles in panel A). VAE = variational autoencoder. AAE = adversarial autoencoder. AE = autoencoder. PCA = principal component analysis. ICA = independent component analysis.

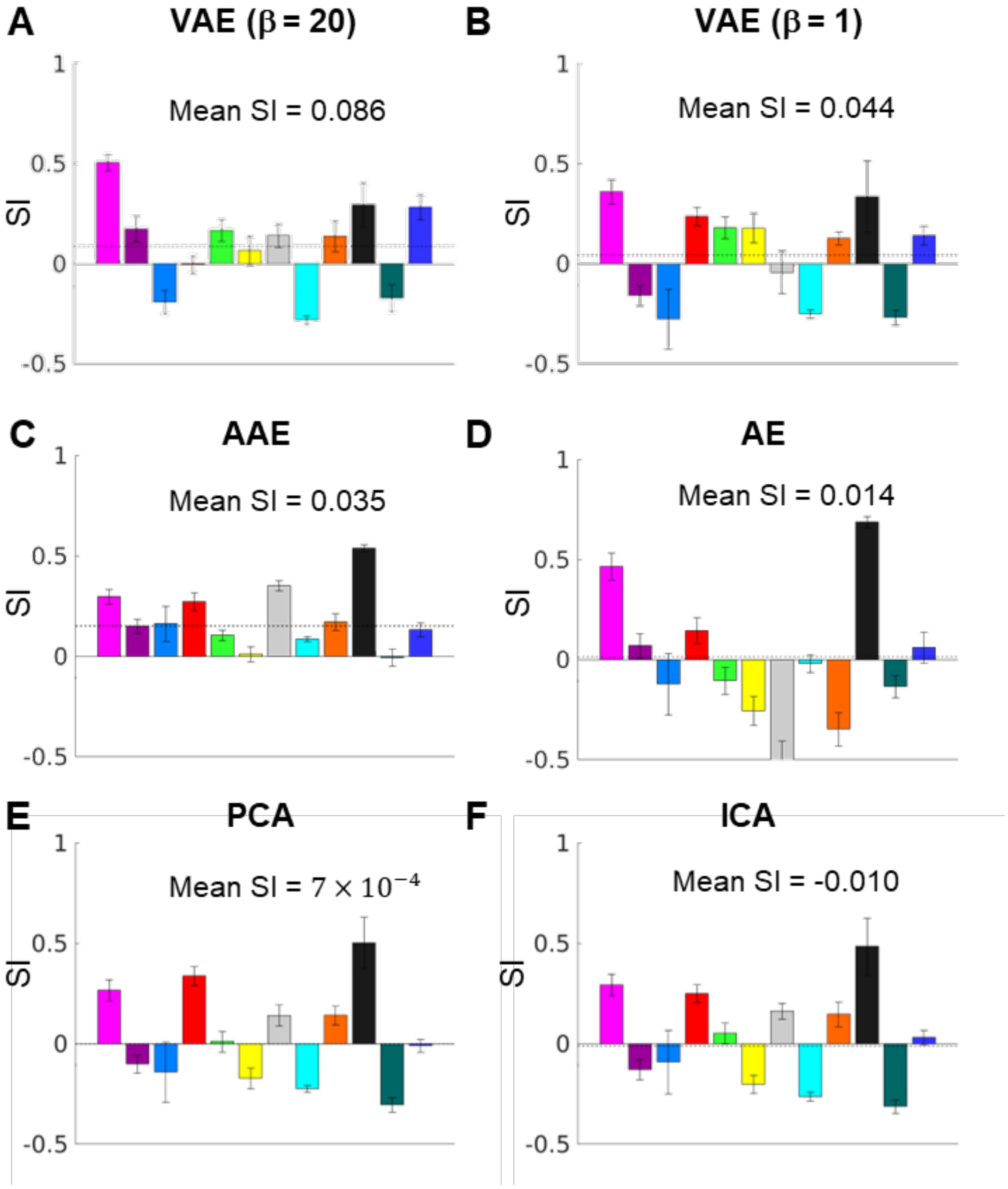

Supplementary Figure D.3-2. *Silhouette index for each functional network based on the Euclidean distance between FC embeddings in the latent space from various dimensionality reduction methods (dimensionality = 2).* VAE = variational autoencoder. AAE = adversarial autoencoder. AE = autoencoder. PCA = principal component analysis. ICA = independent component analysis.

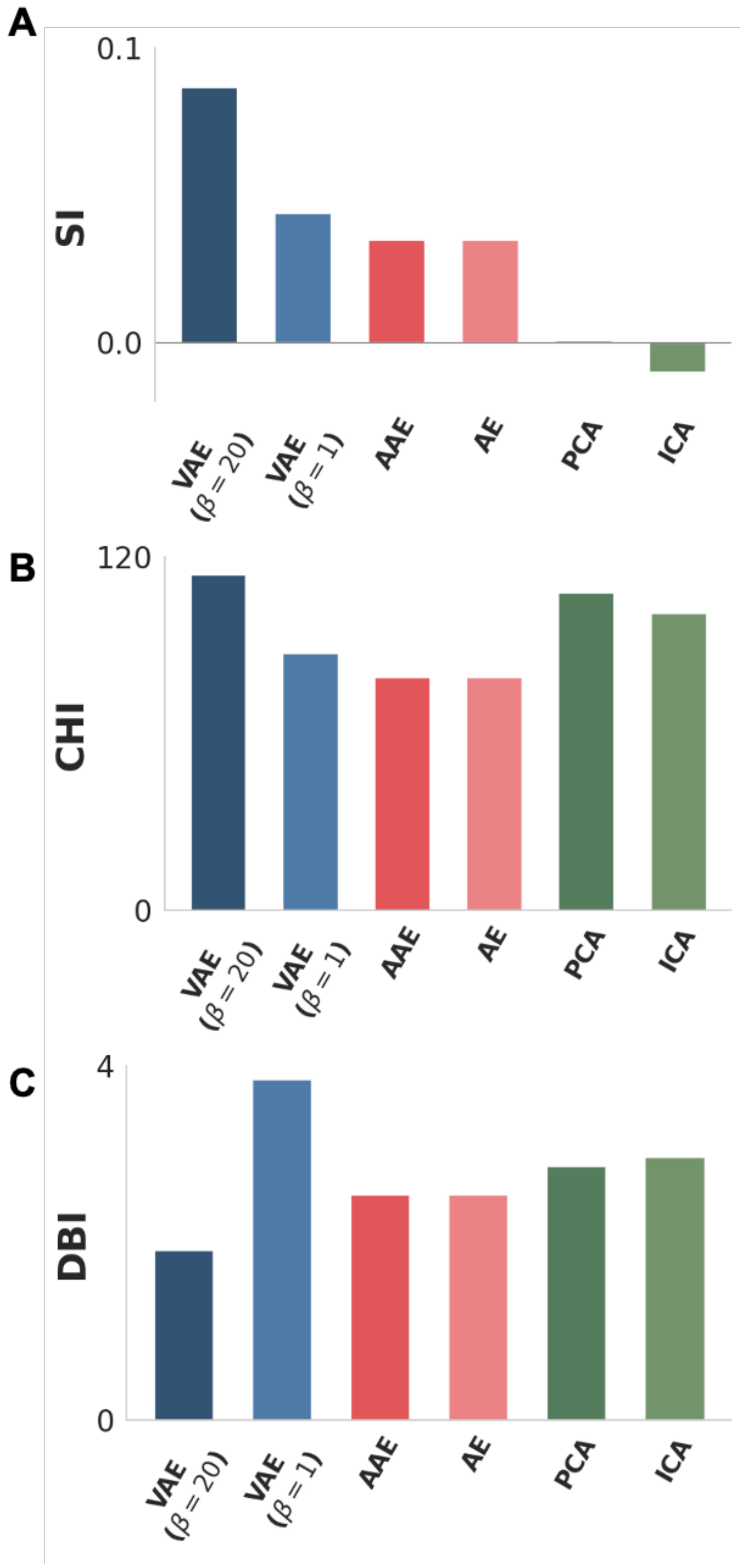

Supplementary Figure D.3-3. *Cluster evaluation metrics based on the Euclidean distance between FC embeddings in the latent space from various dimensionality reduction methods (dimensionality = 2).* A) Average silhouette Index. B) Davies-Bouldin Index. C) Calinski-

Harabasz (CH) Index.VAE = variational autoencoder. AAE = adversarial autoencoder. AE = autoencoder. PCA = principal component analysis. ICA = independent component analysis.

D.4 Reconstruction Performance

Reconstruction performance was calculated with η^2, namely, the fraction of variance in the original FC profile accounted for by variance in the reconstructed FC profile on a point-by-point basis (Cohen et al., 2008). η^2 ranged from 0 (no similarity) to 1 (identical) and is formally defined by:

$$\eta^2 = 1 - \frac{SS_{Within}}{SS_{Total}} = 1 - \frac{\sum_{i=1}^{n} [(a_i - m_i)^2 + (b_i - m_i)^2]}{\sum_{i=1}^{n} [(a_i - \underline{M})^2 + (b_i - \underline{M})^2]}$$

where a_i and b_i represent the values at position i in maps a and b, respectively. m_i is the mean value of the two images at position i, $\frac{a_i + b_i}{2}$. $\underline{M}$ is the grand mean value across the mean image m. Self-connectivity at all positions was excluded. This similarity matrix is sensitive to the difference in a and b in scales and offsets. For convenience, we used the inversely formatted FC profiles from the 2D image in Figure 1A as the reference ground truth data ($\eta^2 > 0.99$) to the original data. to be compared with the reconstructed data from latent representations. We computed the reconstruction performance on the FC profiles from each of the 333 area parcels (Gordon et al., 2016) in the 10 test subjects in the WU120 dataset and the two sessions (Rest1 and Rest2) for the 94 test subjects in the HCP dataset to test the out-of-sample and out-of-distribution generalization, respectively. A good latent representation should reflect a trait-like property of the subject and minimize across-session variation, yet be distinguishable from other subjects. Since the HCP data had two sessions, we calculated two additional reference measure: 1) the η^2 between the original data in HCP Rest1 and HCP Rest2 of the same subject, which provides the noise ceiling of the reconstruction, and 2) the η^2 between each subject to the rest 93 subjects in each session (and then averaged across the Rest1 and Rest2 sessions), which provides the mean baseline.

In the reconstructed FC profile from one example area parcel (parcel 15 in the medial visual cortex, Supplementary Figure D.4-1, a general trend for more detailed variations in reconstruction FC was observed with an increasing number of dimensions (Supplementary Figure D.4-1A). Overall, the mean reconstruction performance across all parcels in each individual was similar across methods. Autoencoder-based latent representations provided better reconstruction performance when the latent representation had only 2 dimensions (Supplementary Figure D.4-1B), while linear methods provided marginally better reconstruction performance at 32 dimensions (Supplementary Figure D.4-1D). In all cases, the reconstruction performance from the latent representations was, on average, higher than the mean baseline, suggesting that the latent representations captured individual-specific features in addition to group-average features. With 32 dimensions, the reconstruction performance was approaching the noise ceiling Supplementary Figure D4-1D). This observation persisted when each subject's average reconstruction performance was normalized to their noise ceiling (Supplementary Figure D.4-2).

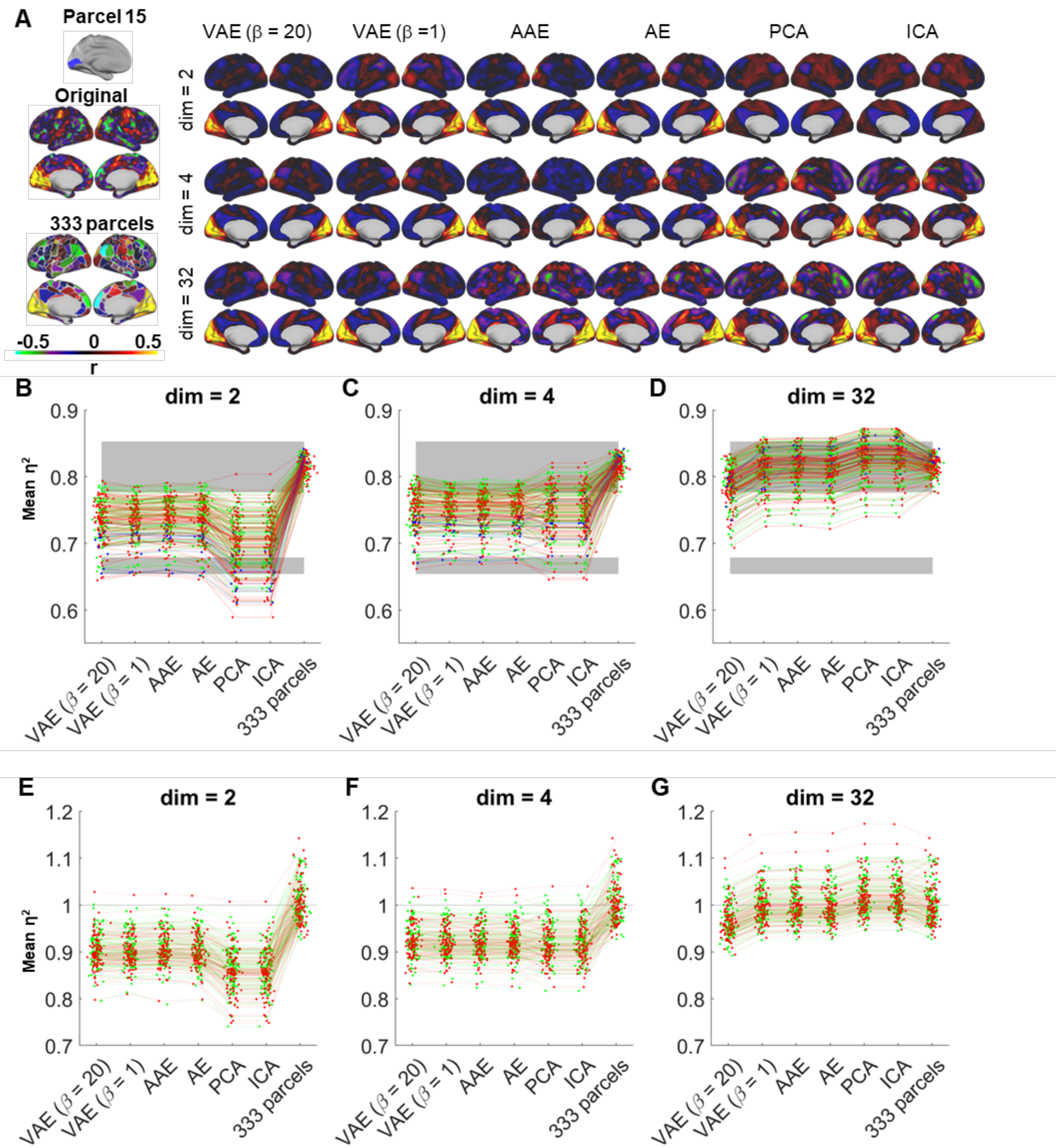

Supplementary Figure D.4-1. *Reconstruction performance comparison across dimensionality reduction methods.* A) Visualization of the original and reconstructed FC profiles from parcel 15 in an example subject (subject 111). B-D) The average reconstruction performance from all parcels for all subjects in the HCP and WU120 test datasets. E-G) Same as B-D but normalized to each subject's noise ceiling. Green line: HCP Rest1, Red line: HCP Rest2, Blue line: WU120. Gray shaded area: mean and standard deviation of the noise ceiling and the mean baseline across 94 HCP subjects. N.B.: 333 parcels always have 333 dimensions and were repeatedly displayed in all three panels as a reference. VAE = variational autoencoder. AAE = adversarial autoencoder. AE = autoencoder. PCA = principal component analysis. ICA = independent component analysis.

D.5 Fingerprinting Performance (intraindividual and interindividual variability)

Fingerprinting performance was calculated for dimensionality 2, 4, and 32 for alternative dimensionality reduction methods.

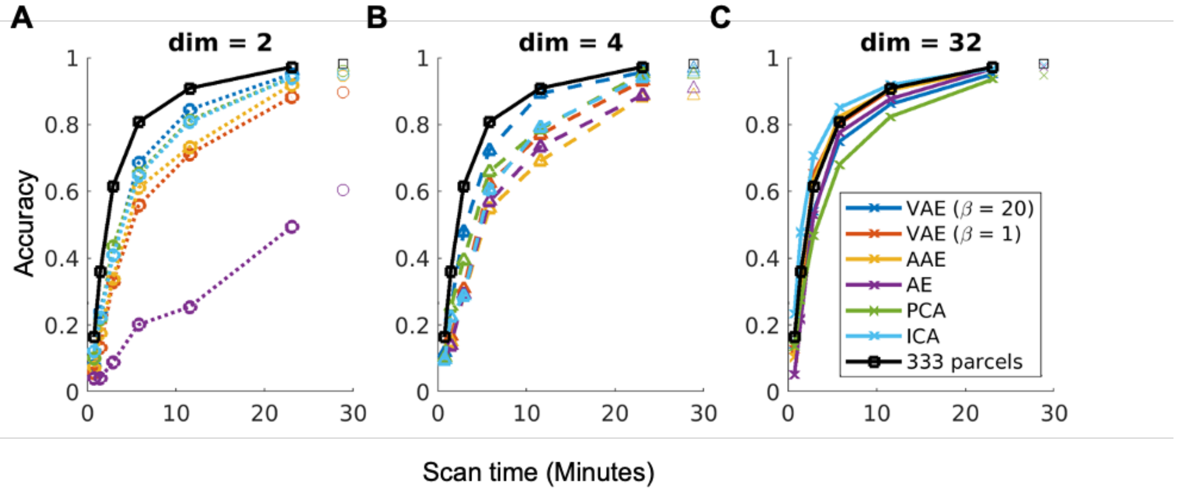

Supplementary Figure D.5 *Fingerprinting performance across alternative dimensionality reduction methods and other latent dimensionality.* The lines show fingerprinting performances from increasing amounts of data in each session, and the isolated markers show the fingerprinting performance from the whole session.VAE = variational autoencoder. AAE = adversarial autoencoder. AE = autoencoder. PCA = principal component analysis. ICA = independent component analysis. The linestyle is consistent within dimensionality = 2, 4, and 32.

D.6 Age Prediction Performance

Age prediction performance was calculated for dimensionality 2, 4, and 32 for alternative dimensionality reduction methods using the 301 sessions of BCP data.

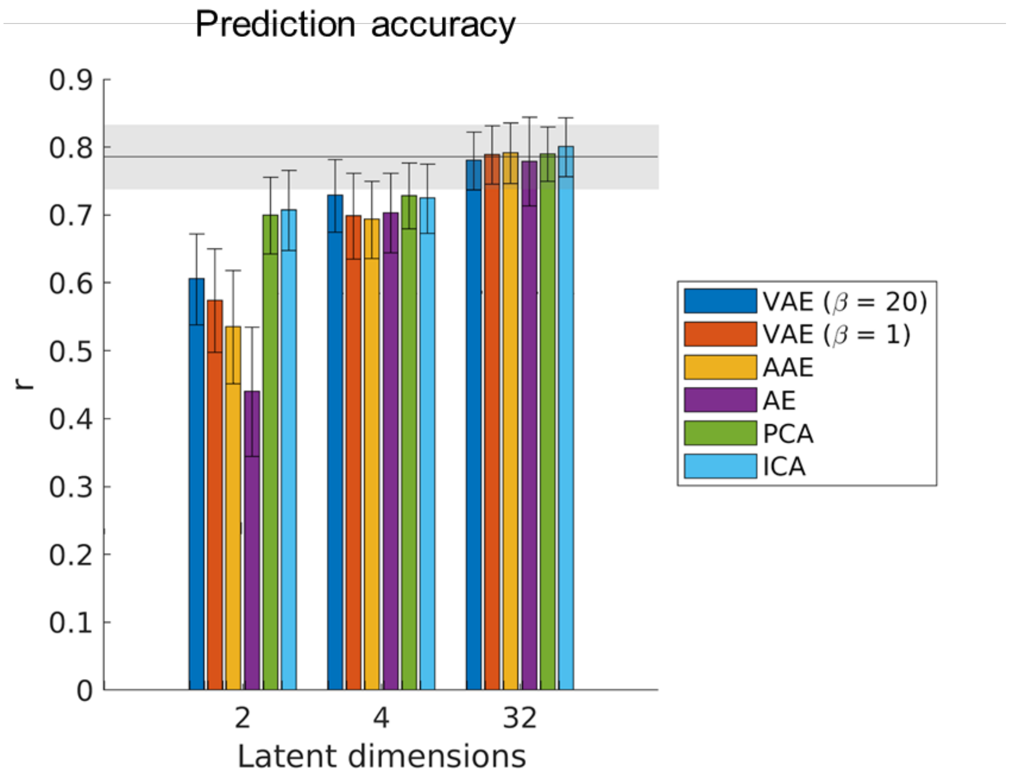

Supplementary Figure D.6 *Age prediction performance across alternative dimensionality reduction methods.* Age prediction accuracy is measured as the Pearson's correlation (r) between the actual and predicted age in years. Error bars show the mean and standard deviation across the 1000 samples. The horizontal line and shaded area show the performance (mean and standard deviation) using features in the parcel space. VAE = variational autoencoder. AAE = adversarial autoencoder. AE = autoencoder. PCA = principal component analysis. ICA = independent component analysis.

Section E. Generalization to Task fMRI

E.1 Task fMRI in HCP

Task-evoked functional fMRI (tfMRI) in the HCP was collected while participants performed the working memory, gambling, motor, language, social cognition, relational processing, and emotional processing tasks in the MRI scanner. Due to the availability of data at hand, we illustrated the generalizability of our results to functional connectivity in tfMRI using the working memory data. The working memory session contains the category-specific representation task and the working memory task, where participants were presented with blocks of trials that consisted of pictures of places, tools, faces, and body parts. Within each run, 4 different stimulus types were presented in separate blocks, and half of the blocks used a 2-back working memory task and half used a 0-back working memory task. Each of the two runs contains 8 task blocks (10 trials of 2.5s each, for 25s) and 4 fixation blocks (15s each). The working memory task was conducted at the first fMRI session with 405 frames per run at TR = 0.72 s (approximately 5 minutes per run for two runs). The acquisition details were identical to the resting-state fMRI acquisitions, apart from run durations. More detailed methods can be found in prior work (Barch et al., 2013). We used the full time series from both runs to construct the

functional connectivity for each subject. The data was first minimally processed and projected to the fsLR-32k standard surfaces using established procedures (Glasser et al., 2013). Additional mitigation of non-neuronal sources of signal was conducted at surface space by regressing out the six realignment parameters, their squares, derivatives, and squares of the derivatives, mean white matter signal, mean cerebrospinal fluid signal and mean global signal of the grayordinates using the DCAN BOLD processing pipeline scripts (https://github.com/DCAN-Labs/dcan_bold_processing) which largely resembled the processing of resting-state data used in the main manuscript. Some differences include that no low-pass filter was applied to the motion parameters before calculation of framewise displacement (FD), and frames with FD>0.3 were interpolated but not removed. The BOLD time series data were then parcellated with the Gordon parcellation (Gordon et al., 2016) and correlated with Pearson's correlation to generate the functional connectivity.

E.2 Comparing Task and Rest FC Embeddings

Each row of the parcellated functional connectivity matrix (333 x 333) was then upsampled to vertex space, a.k.a. 333 x 59412, using the parcel assignment of each vertex in the Gordon parcellation, and passed to the pretrained VAE model to obtain the latent embeddings. The resultant latent embeddings of the 94 HCP subjects are shown in comparison to the latent embeddings from the Rest1 session (Supplementary Figure E.2-1). For the remaining analysis, we remove the parcels from the "None" network, resulting in 286 parcels per subject. We found the general distribution of the parcels in each network was similar in the working memory task compared to the Rest1 session, despite minor differences. Overall, the average silhouette index is lower in the working memory state, suggesting reconfiguration of the networks that deviates from the resting-state network definitions (Supplementary Figure E.2-2).

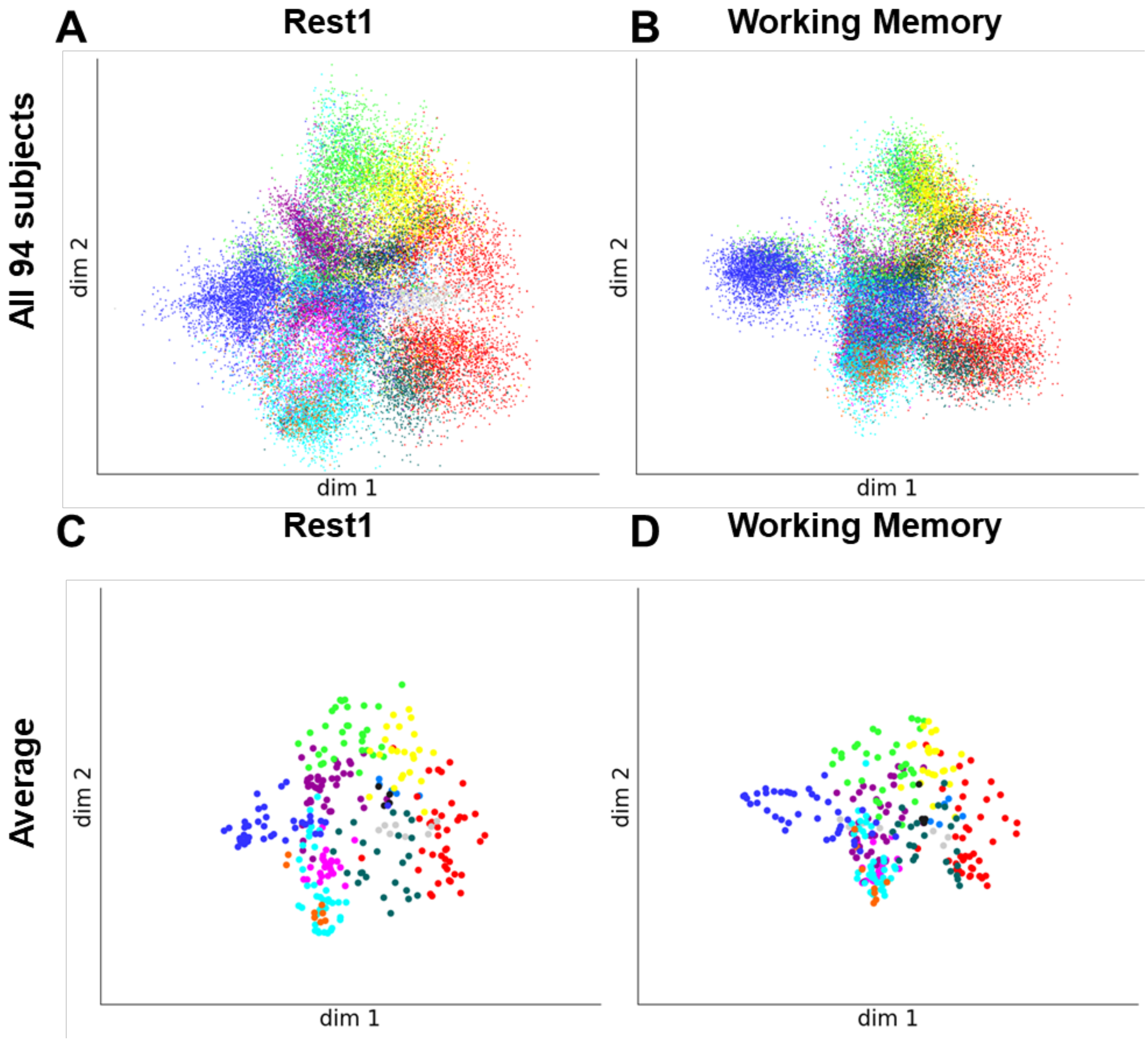

Supplementary Figure E.2-1. *Separation of functional connectivity profiles by functional networks in Rest1 and Working Memory fMRI sessions.* A-B) all 94 HCP subjects and C-D) the average of each parcel across subjects.

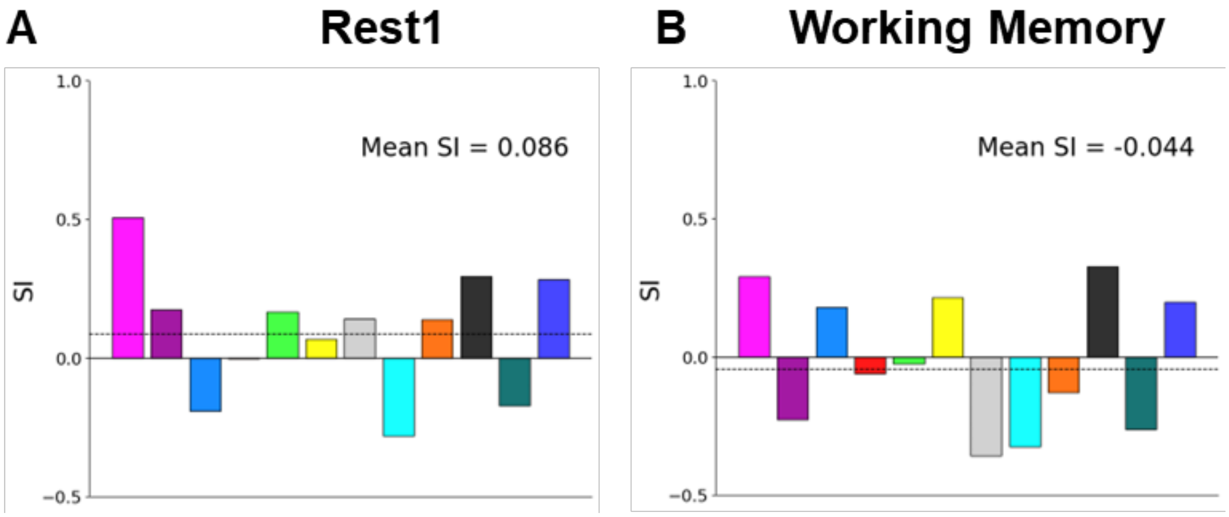

Supplementary Figure E.2-2. *Silhouette index for each functional network based on the Euclidean distance between FC embeddings in the latent space.* A) Rest1. B) Working Memory.

In addition, to test if the distribution of latent embeddings (2862) captured the task-related variance in FC, we plotted a similarity matrix that shows within-state(task/rest) and between-state (task/rest) similarity across subjects (Supplementary Figure E.2-3). We found that the similarity is higher within-state than between states (within Rest1: mean Pearson's r across pairs of 94 subjects = 0.491, standard deviation = 0.065, within Working Memory: mean Pearson's r across pairs of 94 subjects = 0.470, standard deviation = 0.059, between Rest1 and Working Memory: mean Pearson's r across pairs of 94 subjects = 0.372, standard deviation = 0.066). This suggested that the latent embeddings captured the task-related variance in FC, similar to the parcel connectomes (the upper triangle of the 286286 matrix). For parcel connectomes, within Rest1: mean Pearson's r across pairs of 94 subjects = 0.521, standard deviation = 0.061, within Working Memory: mean Pearson's r across pairs of 94 subjects = 0.416, standard deviation =0.069, between Rest1 and Working Memory: mean across pairs of 94 subjects = 0.264, standard deviation = 0.062. We also ran a support vector machine (SVM) classifier with a linear kernel and regularization parameter C = 1 to classify the Rest1 and Working Memory states based on the FC embeddings of all parcels or the FC parcel connectomes using a stratified 10-fold cross-validation. The overall classification accuracy, calculated as the match between predicted and true labels across all 10-folds, was 100% for both the FC latent embeddings at 2 dimensions and the parcel connectome, suggesting that the state-dependent variance of FC is preserved in the latent embeddings.

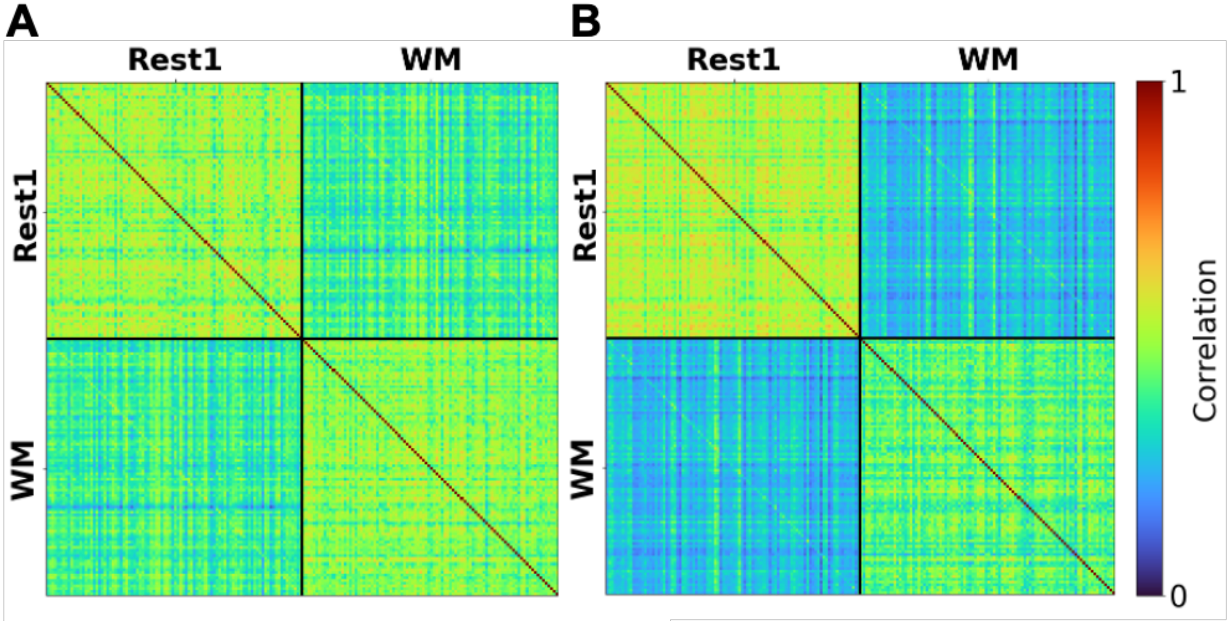

Supplementary Figure E.2-3. Within-state and between-state FC similarity in A) VAE latents, B) 333 parcels.
